# Systems Pharmacology Dissection of the Integrated Treatment for Cardiovascular and Gastrointestinal Disorders by Traditional Chinese Medicine

**DOI:** 10.1038/srep32400

**Published:** 2016-09-06

**Authors:** Wenjuan Zhang, Qin Tao, Zihu Guo, Yingxue Fu, Xuetong Chen, Piar Ali Shar, Mohamed Shahen, Jinglin Zhu, Jun Xue, Yaofei Bai, Ziyin Wu, Zhenzhong Wang, Wei Xiao, Yonghua Wang

**Affiliations:** 1 College of Life Science, Northwest A & F University, Yangling, Shaanxi 712100, China; 2 Center of Bioinformatics, Northwest A & F University, Yangling, Shaanxi 712100, China; 3 College of Life Science, Northwest University, Xi’an, Shaanxi 710069, China; 4 State Key Laboratory of New-tech for Chinese Medicine Pharmaceutical Process, Lianyungang, Jiangsu, 222001, China

## Abstract

Though cardiovascular diseases (CVDs) and gastrointestinal disorders (GIDs) are different diseases associated with different organs, they are highly correlated clinically. Importantly, in Traditional Chinese Medicine (TCM), similar treatment strategies have been applied in both diseases. However, the etiological mechanisms underlying them remain unclear. Here, an integrated systems pharmacology approach is presented for illustrating the molecular correlations between CVDs and GIDs. Firstly, we identified pairs of genes that are associated with CVDs and GIDs and found that these genes are functionally related. Then, the association between 115 heart meridian (HM) herbs and 163 stomach meridian (SM) herbs and their combination application in Chinese patent medicine was investigated, implying that both CVDs and GIDs can be treated by the same strategy. Exemplified by a classical formula Sanhe Decoration (SHD) treating chronic gastritis, we applied systems-based analysis to introduce a drug-target-pathway-organ network that clarifies mechanisms of different diseases being treated by the same strategy. The results indicate that SHD regulated several pathological processes involved in both CVDs and GIDs. We experimentally confirmed the predictions implied by the effect of SHD for myocardial ischemia. The systems pharmacology suggests a novel integrated strategy for rational drug development for complex associated diseases.

Over the past decade, there has been a marked increase in our understanding that there are higher prevalence rates of gastrointestinal disease (GIDs) in patients with cardiovascular disease (CVDs)^1^,^2^ with similar dysfunctional phenotypes, such as rib pain, stomach pain, nausea and vomiting. However, the underlying co-occurrence mechanisms of CVDs and GIDs are unclear, thereby hampering development of drugs for both diseases in humans^3^. In modern Western medicine, usually, it has been observed that cardiovascular diseases have an etiological relationship with gastrointestinal disorders. Several studies have reported that the risk of cardiovascular disease in patients with gastrointestinal disease appears to be far greater than in the general population^4^. Moreover, some gastrointestinal disorders may increase patients’ risk of cardiovascular disease as well. For example, patients with chronic gastrointestinal ischemia have an increased CVDs’ risk and excess mortality^5^. Furthermore, the pathophysiological mechanisms between the two organs would supply realistic treatment for CVDs and GIDs^6^. For example, Iranian traditional physicians have introduced several remedies for heart-stomach association ailments in a previous study^7^. In addition, novel studies demonstrated the close relationship between gastroesophageal reflux disease (GERD) and the development of atrial fibrillation (AF), notably, acid-suppressive therapy is an effective strategy for the management of AF and may help to minimize the use of anti-arrhythmic agents^8^,^9^. However, the pathophysiology or mediators underlying these comorbidities were not clearly elucidated due to the concept of one-gene/one-enzyme/one-function, which viewed these disorders are independent from each other.

Differently, holistic medicine is understood herein including alternative, complementary, and traditional medical practices^6^, sharing consequences in relation to overall health, which may lead to the advancement of therapeutic strategies for complex disorders. In traditional Chinese Medicine, human body is considered as a holistic being in which each organ or each specific physiological part is interrelated. Based on *Huangdi Neijing* (The Yellow Emperor’s Inner Canon)^10^, traditional Chinese medical practitioners diagnose and treat patients beneath the guidance of meridian theory, declaring that the heart and stomach linked through some specific *Jingluo* (meridians)^11^ and they have an interaction effect in terms of both physiological function and pathological basis. Under the *Jingluo* theory, both heart and stomach are located in the epigastrium, and they are separated only by the diaphragm. Furthermore, the relationship between heart and stomach is inter-promoting. As for physiological function, the heart is able to control the blood and vessels and govern the mind, while the stomach transforms food into usable nutrition to ensure the production and storage of blood.

Traditional Chinese Medicine (TCM), which is an integral knowledge system of holistic medicine for disease prevention and treatment based on practitioner’s dynamic observation and practice^12^. TCM is becoming more popular because it is widely available and relatively effective, and has fewer side effects^13^. More importantly, TCM does not focus merely on diseases that are defined by specific pathological changes, but concentrates on the overall functional states of the patients in a holistic fashion^14^. As for synchronic treatment of CVDs and GIDs, the earliest depiction has been documented in *Jingui Yaolue* (Synopsis of Golden Chamber), a classic clinical book written by Zhang Zhongjing, which considered that adjusting the function of spleen and stomach is of great significance for the treatment of heart disease and designed a series of herbal formulae to treat XiongBi (thoracic obstruction) and XinTong syndrome (cardiodynia). For example, the Zhishi Xiebai Guizhi decoction, which consists of Zhishi (Fructus Aurantii Immaturus), Houpu (Cortex Magnoliae Officinalis), Xiebai (Bulbus Allii Macrostemonis), Guizhi (Ramulus Cinnamomi), Gualoushi (Fructus Trichosanthis), is used to strengthen the function of stomach, moreover, it also can be used to treat coronary heart diseases^15^. Besides, some heart Meridian herbs, such as Baihe (*Lilium brownii var.viridulum*), Hehuanpi (bark of *Albizia julibrissin Durazz*), which can nourish the heart, are often added into the formulae to enhance the therapeutic effect for stomach diseases.

Despite the promising effects of TCM in the treatment of CVDs and GIDs, how formulae of heart meridian herbs and stomach meridian herbs work together and what their common targets are is still ambiguous. To clarify molecular mechanisms of herbal drugs for complex related diseases from a system level, the following numerous issues need to be solved urgently: 1) what the relationship is between heart meridian (HM) herbs and stomach meridian (SM) herbs? And how the HM herbs and SM herbs are combined in the treatment of CVDs and GIDs clinically? 2) Which active compounds are involved in the regulatory processes of formulae in CVDs and GIDs treatment? 3) Which targets are modulated by the active ingredients to achieve the therapeutic effect? 4) Which pathologic processes are regulated by the active compounds and herbal medicine to treat CVDs and GIDs simultaneously? 5) Whether the formulae for the GIDs is effective in treating CVDs experimentally? Nowadays, systems pharmacology is an emerging field implicated in the application of network pharmacology and pharmacokinetics evaluation^16^, which clarifies the therapeutic effects and underlying mechanisms of multi-component and multi-target agents^17^,^18^,^19^. In our previous work, we have successfully built an integrated platform of systems pharmacology combined the discovery of bioactive ingredients, prediction of drug targets, exploration of therapeutic mechanisms and revelation of TCM combination rule, etc^20^,^21^,^22^,^23^. For example, under the paradigm, we have investigated the mechanisms for the well-known herbal recipe Compound Danshen Formula (CDF) and the botanical drug licorice^24^,^25^.

In this study, we proposed an integrated systems pharmacology approach to illustrate the mechanisms of action in the systematic combination of different herbs for treating relevant diseases simultaneously. Firstly, we attempted to dissect underlying mechanisms of CVDs and GIDs. Then we explored the association between heart meridian (HM) herbs and stomach meridian (SM) herbs, and evaluate their combination application in Chinese Patent Medicine (CPM). TCM has achieved notable success in treating related complex diseases, here, a classical formula Sanhe Decoction (SHD) was selected as a case study^26^. SHD is a complex system with 7 herbs, including *Lilli Bulbus, Salvia Miltiorrhiza, Alpinia Officinarum Rhizoma, Amomum Villosum, Santail Albi Lignum, Lindera Aggregata* and *Cyperus Rotundus*. Among these herbs, *Radix Salvia* and *Lilli Bulbus* belong to heart meridian; *Alpiniae Officinarum Rhizome* and *Fructus Amomi* belong to stomach meridian; *Santail Albi Lignum* belongs to both HM and SM; *Rhizoma Cyperi and Radix Linderae* are assigned to other meridians. At first, Sanhe Decoration (SHD) has been applied to treat GIDs such as chronic gastritis, epigastric pain and peptic ulcer, and the stomach meridian herbs in the formula have been reported to treat stomach diseases in previous study. For example, *Alpiniae Officinarum Rhizome* and *Fructus Amomi* have been validated to strengthen the stomach and relieve stomach ache^27^,^28^. In addition, modern pharmacological studies have proven that *Radix Salvia* could treat CVDs by enhancing myocardial anti-hypoxia capacity, increasing myocardial contraction and improving cardiac function^29^, thus we inferred that Sanhe Decoction may be applied for the treatment of both CVDs and GIDs.

An integrated systems pharmacology approach was used to depict the holistic healing of SHD for cardiovascular-gastrointestinal diseases from a molecular to holistic level. Briefly, as shown in Fig. 1, we screened the biochemical compounds by *in silico* ADME system and predicted the potential related targets of these compounds by weighted ensemble similarity (WES) method^30^,^31^. The obtained targets were mapped onto relevant databases to find out their corresponding pathways of CVDs and GIDs. Furthermore, network construction, pathway enrichment analysis and tissue location analysis were performed to illustrate the molecular mechanisms of SHD on CVDs and GIDs holistically. Finally, we administered SHD to LAD occlusion model rats and investigated the effect of SHD on apomorphosis and necrosis of cardiac muscle cells and the activity change of superoxide dismutase (SOD), creatine kinase (CK), cyclic adenosine monophosphate (cAMP) and cardiac troponin I (cTnI). These results further supplied the *in vivo* experimental evidence to validate the therapeutic effect of SHD for CVDs that were predicted in the systems pharmacology. In summary, our systems pharmacology approach would provide guidance for complicated related disease treatment and new drug development.

## Results

### The closeness analysis between genes of CVDs and GIDs

We collected 288 and 353 genes associated with CVDs (31 types) and GIDs (13 types), respectively. The detailed information of these genes were listed in supplementary Tables S1 and S2, respectively. As shown in Table 1, 47 shared genes of CVDs and GIDs were identified. If two diseases share a large number of disease genes, the disease pairs become more comorbid and they are closely associated^32^. Therefore, shared genes of CVDs and GIDs could provide novel target potential for the treatment of CVDs and GIDs. Among these 38 genes, many targets have been proved effective in the clinical practices, for example, the regulation of gene angiotensin I converting enzyme (ACE) has therapeutic effect on chronic heart failure and gastric cancer^33^,^34^. ACE blockers are now first-line treatments for hypertensive target organ damage. In addition, ACE is located at the upstream of renin-angiotensin system pathway. Renin-angiotensin system blockade exerts potent anti-atherosclerotic effects, which are mediated by their antihypertensive, anti-inflammatory, anti-proliferative, and oxidative stress lowering properties^35^. Moreover, the renin-angiotensin system pathway plays a pivotal role in regulating the blood volume and systemic vascular resistance, which together influence cardiac output and arterial pressure. ACE inhibitors are often used to prevent angiotensin I and angiotensin II from binding to blood vessels and causing vasoconstriction, since angiotensin II is a strong hormone in renin-angiotensin system pathway, and can act directly on blood vessels to cause blood pressure increases. Gene Nitric oxide synthase 2 (NOS2) is associated with both heart failure and gastric ulcer, which is involved in inflammatory response and its expression could prevent harmful processes to human^36^,^37^. Specially, the transcription regulation of NOS2 depends on the mediation of specific transcription factors, such as CREB, NF-κB and C/EBP81^38^, thus playing the therapeutic effects.

**Figure 2:**
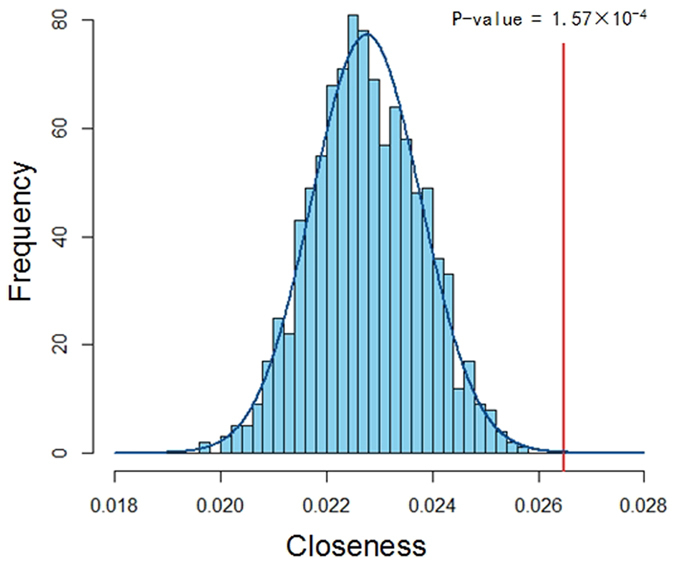
The closeness correlation of gene pair between CVDs and GIDs.

Though shared genes of CVDs and GIDs are not so abundant, previous study found that if pairs of genes are closely related in PPI network, these pairs of genes are significantly correlated^39^. To assess the correlation of pairs of genes between CVDs and GIDs, we illustrate their closeness in PPI network. According to the formula (1), the obtained closeness between genes of CVDs and GIDs is 0.0264 by the 10,000 times of randomization (Fig. 2). To evaluate the statistical significance between the actual distance and those of random counterpart, Z test is carried out and the significant difference is defined as P < 0.01. Compared with the random-selected two proteins from PPI network, two group genes associated with CVDs and GIDs show significantly close linkage relevance (ultimate nearness = 1.57 × 10^−4^ (p < 0.01)). These results above indicated that genes associated with CVDs and GIDs are close related.

### Correlation of the heart meridian (HM) and stomach meridian (SM) herbs

**Figure 3:**
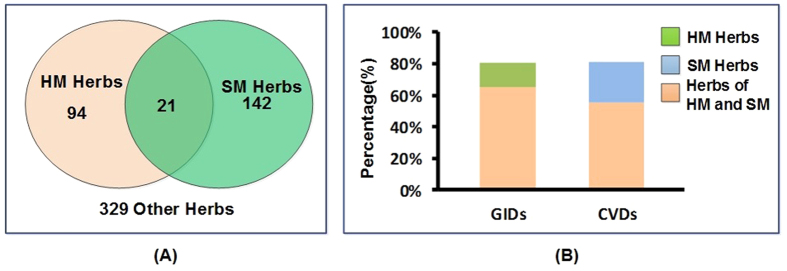
Correlation of the HM and SM herbs. (**A**) The relationship of HM and SM Herbs. (**B**) Distribution of these herbs in CPM for CVDs and GIDs.

115 and 163 herbs that belonged to heart meridian and stomach meridian were collected from Chinese Pharmacopeia, respectively (The herbs were presented in Supplementary Tables S3 and S4, respectively). The Venn diagram illustrated that 21 herbs belonged to both HM and SM (Fig. 3(A), the detailed information of common herbs was listed in Table 2). Specifically, common herbs belonged to both HM and SM meridians includes licorice, *Santalum Albi Lignum*, and dried *Ginger* and so on. In traditional medicine, licorice has been widely used for treating cardiovascular diseases^40^. In fact, licorice is a common herb with desirable pharmacological effects on inhibiting inflammatory processes of blood vessels, preventing atherosclerosis, reducing plasma lipid levels, and decreasing systolic blood pressure^40^,^41^, thus exerting cardio-protective activities^42^,^43^,^44^. Also, licorice has been used as an anti-peptic ulcer agent^45^, and its antiulcer and mucosal protective actions have been confirmed by numerous clinical trials and animal experiments^46^,^47^,^48^. *Santalum Albi Lignum* showed good curative effect in the treatment of anginal attacks^49^ and ulceration *in vivo*^50^. The dried *Ginger Rhizome* has been used as an herbal therapy for treating cardiovascular diseases such as atherosclerosis, hyperglycemia and hyperinsulinemia^51^,^52^. Moreover, the active constituents of *Ginger Rhizome* are effective in alleviating the symptoms of gastrointestinal illnesses^53^. In addition, the Fisher’s test exhibited the significant correlation of the HM and SM herbs (P < 0.01).

Furthermore, to explore the clinical application of the herbs of HM and SM, we analyzed the distribution of these herbs which are used in Chinese Patent Medicine (CPM) for CVDs and GIDs (The list of these CPM are displayed in Tables S5 and S6, respectively). As shown in Fig. 3(B), 81.01% (128/158) herbs of heart meridian appeared in the CPM which are used to treat CVDs, and 68.75% (88/128) herbs of stomach meridian are also found in these CPM. Interestingly, in terms of the CPM for stomach diseases, there was 80.72% (67/83) botanic drugs of stomach meridian, among these TPM, 80.60% (54/67) herbs of heart meridian appeared simultaneously. We concluded that herbs belonging to heart and stomach meridians were highly overlapped in CPMs for the treatment of heart and stomach diseases, indicating that the combination of heart and stomach herbs may be a successful strategy to treat CVDs and GIDs.

### Screening active ingredients of Sanhe Decoction

Sanhe Decoction consists of 7 herbs, *Radix Salvia* and *Lilli Bulbus* belong to heart meridian; *Alpiniae Officinarum Rhizome* and *Fructus Amomi* belong to stomach meridian; and *Santail Albi Lignum* belongs to both HM and SM; *Rhizoma Cyperi and Radix Linderae* are assigned to other meridians. By *in silico* ADME model, 59 compounds were predicted as the potential active compounds with appropriate pharmaceutical properties, which accounted for 8.9% of the total compounds, including 32 phenanthrenequinone compounds, 10 terpenoid compounds, 8 flavonoid compounds, 4 ligand compounds and 2 coumarin compounds. The ADME parameters and structural information of these 59 compounds are shown in Table 3. Among 59 predicted ingredients, 6 compounds converted by intestinal microbes. M1 (Luteolin) is converted into baicalein 6-methylether^54^, M2 (Quercetin) is metabolized to myricetin, 8-hydroxy quercetin and 3′-O-methylquercetin by biotransformation^55^, M8 (Galangin) is metabolized to kaempferol. M31 (Cryptotanshinone) is able to be biotransformed to three new products, (3R,15R)-3-hydroxycryptotanshinone, (3S,15R)-3-hydroxycryptotanshinone, and (4S,15R)-18-hydroxycryptotanshinone^56^. M45 (Neocryptotanshinone) could be convert into CT, TIIA, TIIB and TI, and probably DH-TI stepwisely^57^. The metabolites of M52 (Tanshinone IIA) are formed through hydroxylation and dehydrogenation, including tanshinone IIA, hydroxytanshinone IIA, przewaquinone A and dehydrotanshinone IIA^58^.

There are 41 ingredients in *Radix Salviae* that exhibit valuable therapeutic effects for CVDs and GIDs. For instance, M1 (Luteolin) is a representative compound of SHD that shows cardio-protective effects against ischemia/reperfusion (I/R) injury by reducing necrosis and apoptosis in rat cardiomyocytes^59^. Additionally, Luteolin is also a potential alternative in the treatment of GIDs^60^. M52 (Tanshinone IIA) is one of the key active ingredients of *Radix Salviae* and it protects cardiac myocytes against oxidative stress-induced apoptosis *in vivo*^61^. Tanshinone IIA plays important roles in ischemic heart diseases, especially in reduction of myocardial infarct size and decrease of myocardial consumption of oxygen^62^.

Bioactive ingredients in *Alpinia Officinarum Rhizoma* are M2 (Quercetin), M3 (Isorhamnetin), M04 (Kampferol), M08 (Galangin), M09 (Medicarpin) and so forth. Quercetin is a typical flavonol-type flavonoid with strong antioxidative and cardioprotective properties, thus preventing cardiovascular diseases^63^. M4 (Kaempferol) was reported to protect against coronary heart disease, and a person with higher kaempferol intakes would have the lower incidence of cardiovascular diseases^64^,^65^. M8 (Galangin) has promising effect in the treatment of cardiovascular by reducing lipid peroxidation^66^. In addition, M3 (Isorhamnetin) may be a potential candidate in the treatment of gastric cancer^67^.

6 potential active compounds are identified in *Rhizoma Cyperi*, among them, M16 (Sugeonyl acetate) is the potential effective ingredient for treating CVDs and GIDs pharmacological and biological activities^68^. In *Lilli Bulbus*, 3-Demethylcolchicine is predicted to reduce the risk of cardiovascular diseases (CVDs). High intake of M54 (Vitamin E, in *Fructus Amomi*) through diet or supplements decrease occurrence of cardiovascular diseases and stomach cancer^69^,^70^. M59 (Boldine, *Radix Linderae*) is a major alkaloid with antioxidant activity and anti-inflammatory effect^71^, therefore it is the therapeutic agent for CVDs.

### Compound-Target network for GIDs (C-T1)

**Figure 4:**
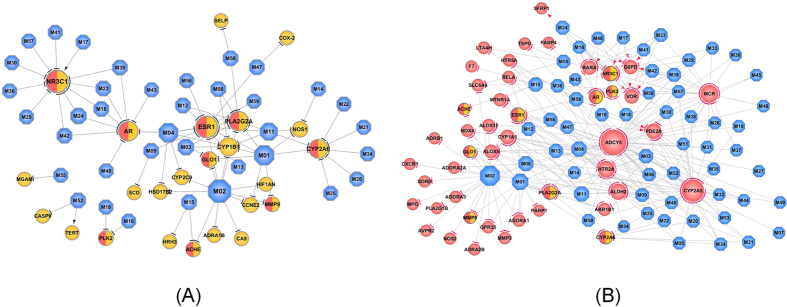
Therapeutic compound-target network. (**A**) Therapeutic compound-target network of GIDs. (**B**) Therapeutic compound-target network of CVDs. The hexagon nodes represent compounds, and circles are targets. Node size is proportional to its degree. Arrows indicate activation and T-arrows represent inhibition of the action mode of compound and target interaction.

We identified proteins that could be potential therapeutic targets of heart and stomach disorders and elucidated the mechanisms of SHD from a systematic level. A compound-target network was constructed to interpret the therapeutic mechanisms of the Sanhe Decoction for GIDs (Fig. 4 (A)). 80 interactions are generated by modulating 24 stomach related targets through 37 compounds. In a network, the node degree (the number of connections or edges the node has to other nodes) is one of the most basic quantitative properties and nodes with highly connections (high degree) are referred to as hubs^72^. Of the 24 targets, 7 proteins possess degree larger than 4 under an average value of 3.3, therefore, these candidate targets participating in more interactions than other proteins are the hubs in this C-T1 Network.

In the network, ESR1 (Estrogen Receptor) was the target with the highest degree (DD = 11), followed by NR3C1 (Glucocorticoid receptor, DD = 10), PLA2G2A (Phospholipase A2 group IIA, DD = 8), AR (Androgen Receptor, DD = 8), CYP2A6 (Cytochrome P450 2A6, DD = 7), CYP1B1 (Cytochrome P450 1B1, DD = 6) and etc. Evidence suggests that the potential active compounds in SHD can act on these targets, thus contributing to therapeutic effect on stomach diseases. For example (as shown in Tables 4), (1) Estrogen receptor (ER) mediates estrogenic activity in several organs, including those in the stomach. The estrogen receptor-α could be expressed in cardiomyocytes and plays an acute cardioprotective role in ischemia reperfusion injury^73^,^74^. Besides, the clinical significance and prognostic effect of ER-β was evaluated, ER-β has a protective effect against gastric cancer, which indicating that hormone therapy may be a useful new strategy for the treatment of gastric cancer^75^. (2) The activity of PLA2G2A (by M1 (Luteolin), M2 (Quercetin), M3 (Isorhamnetin), M4 (Kaempferol), M8 (Galangin), M11 (Chryseriol), M12 (8-Isopentenyl-kaempferol), M58 (Nubigenol)) suppresses progression or metastasis of human gastric cancer^76^. Elevated expression of PLA2G2A might inhibit progression of gastric cancer through increased release of arachidonic acid, thus preventing bacteria invading during inflammation process^77^. (3) Androgen receptor (AR) plays an important role in gastric cancers and is involved in tumor progression^54^. Especially, AR promotes esophageal cancer cell migration and proliferation via matrix metalloproteinase 2^78^. In Sanhe Decoction, M4 (Kaempferol), M16 (Sugeonyl acetate), M23 (Przewalskin A), M24 (Przewalskin B), M39 (Microstegiol), M43 (Miltirone II) and M48 (Salvilenone I) were identified to block AR, highlighting therapeutic effects of on the stomach diseases.

Interestingly, though proteins such as Glyoxalase I (GLO1, DD = 3), Nitric Oxide Synthase (NOS1, DD = 3), Matrix metalloproteinase-9 (MMP9, DD = 2) and Acetylcholinesterase (ACHE, DD = 2) do not have high topological properties, they are all the therapeutic targets of stomach diseases. For example, detoxifying enzyme GLO1 is a potential therapeutic target of human gastric cancer^79^, it was inhibited by M1 (Luteolin), M2 (Quercetin) and M4 (Kaempferol) (in herbs *Salvia miltiorrhiza; Alpinia Officinarum Rhizoma; Rhizoma Galangae* and *Lindera Aggregate*), therefore enhancing the therapeutic effect for GIDs by the synergistic effect in Sanhe Decoction. In addition, the nitric oxide synthase (NOS) family of enzymes synthesize NO in endothelial cells^80^, which augments the generation of reactive oxygen species by mitochondria, and thereby triggering mechanisms of cell survival or death^81^. NO is a mediator of vasodilatation, platelet aggregation, and regulates various cellular functions in immune and inflammatory processes^82^. Besides, endogenous NO derived from NOS contributes to mucosal defense against gastric damage^83^. MMP-9 has been shown to be one of the important enzymes in the invasion and metastatic cascade of gastric cancer^8^,^84^,^85^, thus the MMP9-inhibitor agent is the effective treatment for GIDs. In summary, the action mechanism for SHD is most probably due to that the potential active compounds target at multiple proteins in the biological network, and these targets interacted with each other so that play therapeutic effect as a whole for GIDs.

### Compound-Target network for CVDs (C-T2)

The network of compounds and targets for CVDs is shown in Fig. 4(B). And the interaction modes of the compounds and targets are depicted as in the materials and methods of ‘Predicting the mode of action of drugs’. As shown in Fig. 4(B), there are 294 compound-target interactions in the network, and 98% of the targets were inhibited by the potential compounds in SHD. The targets with high degree (DD > 3) were listed in Table 5. Among these targets, Adenylate cyclase type V (ADCY5, DD = 47) is the protein with highest degree, that is to say that, ADCY5 could interact with the most nodes in the network, which plays a key role in regulating the network as the hub target. Moreover, followed by Cytochrome P450 2A5 (CYP2A5, DD = 35), Mineralocorticoid receptor (NR3C2, DD = 20), 5-hydroxytryptamine receptor 2A (HTR2A, DD = 18) and so forth, which are potential therapeutic targets for CVDs. Among these targets, Adenylate cyclase (AC) is the keystone of sympathetic transmission in β-AR signaling in myocardium, and β-AR plays a role in the development of aging cardiomyopathy^86^. The inhibition of ADCY5 prevents myocardial apoptosis potentially by enhancing resistance to oxidative stress and apoptosis^87^. NR3C2 (Mineralocorticoid Receptor) antagonists improve outcomes in patients with chronic heart failure caused by LV systolic dysfunction and hypertension by minimizing the cardiovascular damages^88^,^89^. In Sanhe Decoction, 20 molecules such as M18 (3α-hydroxytanshinoneIIa), M23 (Przewalskin a), M26 (Przewaquinone c) and M28 (Tanshinaldehyde) can inhibit NR3C2 activity, highlighting their therapeutic effect for CVDs. Most drugs used clinically are metabolized by cytochromes P450, specifically, CYP2A5 plays an important role in the regulation of oxidative stress in mice^90^. Intriguingly, the therapeutic target CYP2A5 regulates platelet activation in blood to reduce coronary heart disease in animal models^91^. Notably, CYP2A5 was predicted to be inhibited by 18 molecules in herbs *Salvia Miltiorrhiza* and *Alpinia Officinarum Rhizoma*, further highlighting the synergistic effect in treatment of CVDs. All these results above indicate that the CVDs treats the cardiovascular disease based on the synergistic interactions of different components.

### Target-Pathway network

**Figure 5:**
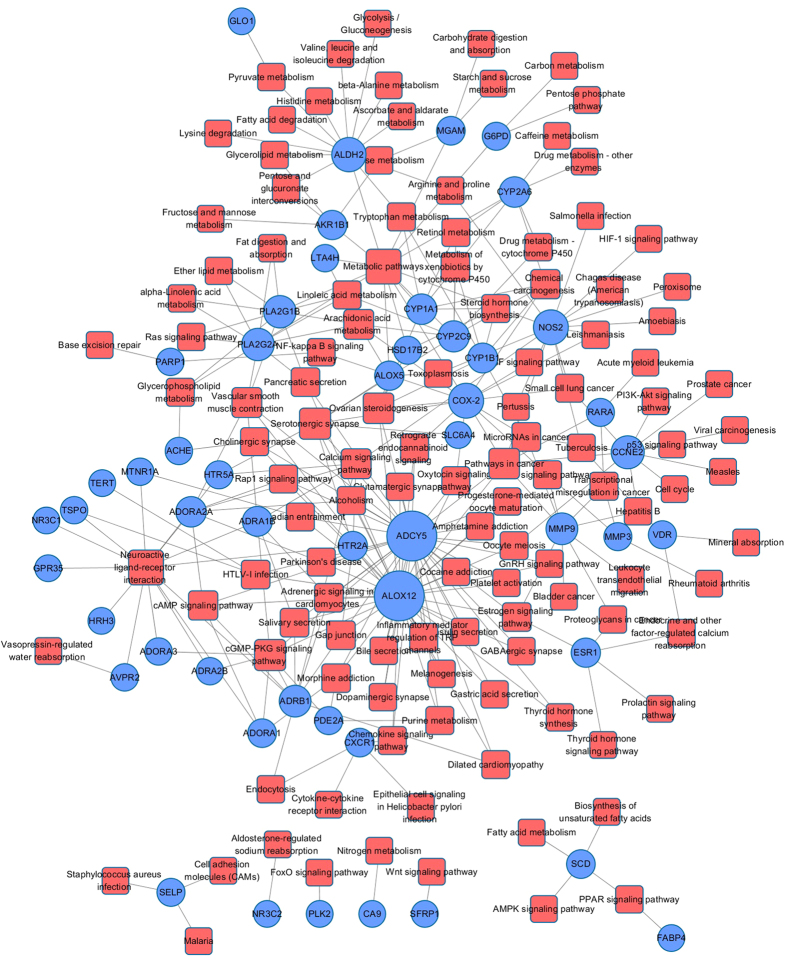
Target-Pathway network of active ingredients in Sanhe Decoction for CVDs and GIDs. Circles are targets and squares are pathways.

Different network regions may underlie different biological pathways, processes or cellular localizations. Drugs not only regulate their related targets, but also affect various metabolic enzymes and downstream proteins, as well as pathways related to the specific disease^24^. To understand the therapeutic mechanisms of SHD, we mapped the predicted targets onto their related pathways extracted from KEGG database (www.genome.jp/kegg)^92^ and generated a bipartite graph of T-P Network (Target-Pathway Network, Fig. 5), in which a compound and a signal pathway were linked if the compound targets on the proteins appeared in the signal pathways. Target-Pathway network consists of 171 nodes (51 targets and 120 pathways) and 294 edges after discarding 8 target proteins not participating in any pathways. Clearly, these pathways are linked with target proteins intensively, nearly 80% target proteins (40/51) are mapped in multiple pathways, indicating that these targets may regulate the interactions and cross-talk between multiple pathways. Similarly, major pathways (72/120) are also modulated through more than one target protein, and many of them have been proved as suitable therapeutic pathways for CVDs and GIDs, such as cGMP-PKG signaling pathway (hsa04022), calcium signaling pathway (hsa04020), cAMP signaling pathway (hsa04024), vascular smooth muscle contraction (hsa04270) and Arachidonic acid metabolism (hsa00590). The cGMP-PKG signaling pathway plays a significant role in cardioprotection by monitoring cell death and maintaining intracellular acidosis^93^. Calcium signaling pathway could mediate autophagy and apoptosis, and Calcium (Ca^2+^) ions regulate muscle contraction, electrical signals which determine the cardiac rhythm and cell growth, exerting pleiotropic effects on cardiovascular cells^94^,^95^. Vascular smooth muscle contraction also plays an important role in the regulation of CVDs, there is due to that proliferation of intimal vascular smooth muscle cells could induce the development of atherosclerosis. Therefore, inhibiting the proliferation of vascular smooth muscle cells is an effective way to control the process of CVDs^24^.

**Figure 6:**
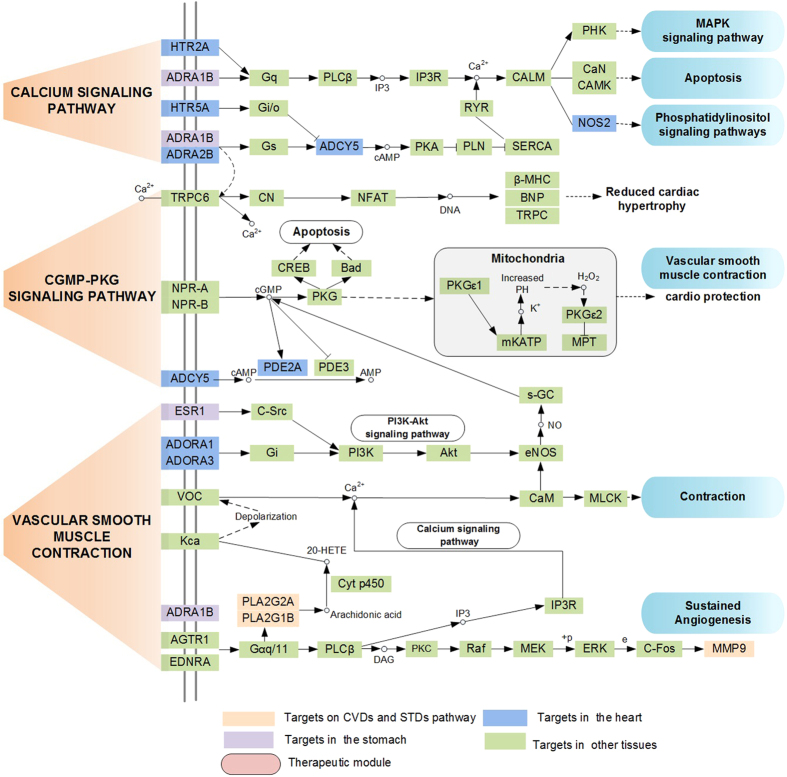
The representative cardiovascular-gastrointestinal disease pathway and therapeutic modules.

Subsequently, based on the current knowledge of cardiovascular-gastrointestinal disease pathology, we mapped these targets into the DAVID database (https://david.ncifcrf.gov/)^96^ to identify the significantly overrepresented incorporated “cardiovascular-gastrointestinal disease pathway”. 51 targets were involved in the most highly related pathways associated with CVD and GIDs in three biological processes, which may provide basis for CVDs and GIDs treatment strategies as well. The functional annotation clustering analysis further illustrated that, as shown in Fig. 6, the enriched pathways under different biological processes are mainly enriched in several modules, including inflammation, contraction, cardiac hypertrophy, cardio protection and apoptosis.

#### Cardiac contraction process

Calcium signaling pathway and vascular smooth contraction pathway play important roles in the contraction function, which are disturbed by the active ingredients in Sanhe Decoction. As shown in Fig. 6, M2 (Quercetin) could reduce ADRA1B protein levels in the upstream pathways-vascular smooth muscle contraction pathway and calcium signaling pathway. Specially, ADRA1B participates in the control of vascular tone and plays an important role in regulating systolic blood pressure levels^97^, thus treating CVDs. In addition, PLA2G1B protein is the key gene in the control of contractions^98^, and it serves as a distinct target in the regulation of active lipid metabolites that promote inflammatory metabolic diseases including CVDs, such as atherosclerosis and hyperlipidemia^99^. Moreover, eight molecules (M1 (Luteolin), M2 (Quercetin), M3 (Isorhamnetin), M4 (Kaempferol), M5 (1,2,5,6-tetrahydrotanshinone), M6 (Isoimperatorin), M8 (Galangin), M9 (Medicarpin)) in SHD serve as activators of ADCY5, which is involved in vascular smooth muscle contraction^100^. In addition, calcium channel blockers are promising interventions for preventing cardiovascular events by lowering blood pressure^101^. The cyclic nucleotides signaling regulates cardiac function, therefore, it may provide novel treatments to improve heart function for hypertrophy and heart failure^102^. The central role of cyclic nucleotide phosphodiesterase (PDE) regulates the activities of the cardiovascular system^103^.

#### Inflammation process

NOS2 plays a central role in the inflammatory reactions and expresses protective effects against detrimental damage^104^. Regulation of NOS2 gene transcription appears to be the primary mechanism of action of cAMP, and whether it is stimulatory or inhibitory depends on the regulation of transcription factors including CREB, NF-κB and C/EBP^105^. Taken together, herbal ingredients in SHD mainly target on proteins ADORA1 and ADORA3, thereby regulating pathways such as PI3K-AKT pathway^106^ and MAPK pathway to reduce stomach inflammation. Besides, controlling of chronic infection in stomach is appropriate for its local effects, at the same time, proving the efficacy in the prevention of CVDs^107^. Therefore, Sanhe Decoction might provide an efficient system for preventing/treating both CVDs and GIDs by the anti-inflammation effect.

#### Apoptosis Process

Analogously, the activation of cGMP-PKG signaling pathway is essential for inhibiting the apoptosis and protecting the heart from ischemia/reperfusion injury^93^. And 13 herbal ingredients like M2 (Quercetin), M8 (Galangin) and M10 (Dehydrotanshinone II A) have been identified to regulate the cGMP-PKG signaling pathway in cardiac ischemia, thereby exhibiting protective effects against heart injury^93^. In addition, the majority of active ingredients in Sanhe Decoction targeted on proteins such as PLA2G1B, ADCY5 and ADORA1, and thereby exhibiting anti-apoptosis effects by regulating the cGMP-PKG signaling pathway^108^,^109^.

cAMP is a unique second messenger, which plays an anti-apoptotic role in protecting rat cardiac myocytes^110^, the changes in the phosphorylation of individual substrates of cAMP-dependent protein kinase is beneficial for the CVDs^111^. Additionally, the alterations of cAMP-mediated signaling pathway have different roles in the pathophysiology of the dilated cardiomyopathy^111^. Therefore, we speculate that herbal medicines of Sanhe Decoction probably mediates these pathways to exhibit the anti-apoptosis, thereby providing an effective strategy to treat CVDs and GIDs systematically. Adenylyl cyclase activity regulates heart failure due to myocardial infarction (MI) in rats^112^, especially, the soluble adenylyl cyclase plays an important role in apoptosis in coronary endothelial cells and cardiomyocytes^113^. Moreover, and adenylyl cyclase also regulates the function of gastrointestinal tract^114^. Therefore, the inhibition of adenylyl cyclase activity is an effective strategy to treat cardiovascular disease and gastrointestinal disorders.

In summary, all the pathways were interdependent with each other through the potential active compounds, which further indicates that SHD can exert synergistic influences on different pathways. In addition, a compound may target multiple proteins involved in multiple pathways, which indicates that the multiple targets of SHD could act on multiple biological processes to treat CVDs and GIDs effectively. We conclude that SHD probably mediates multiple pathways to promote the cardiac contractility properties, display anti-inflammatory and anti-apoptosis properties, and thereby exhibiting synergistic effect for CVDs and GIDs.

#### Target tissue location

**Figure 7:**
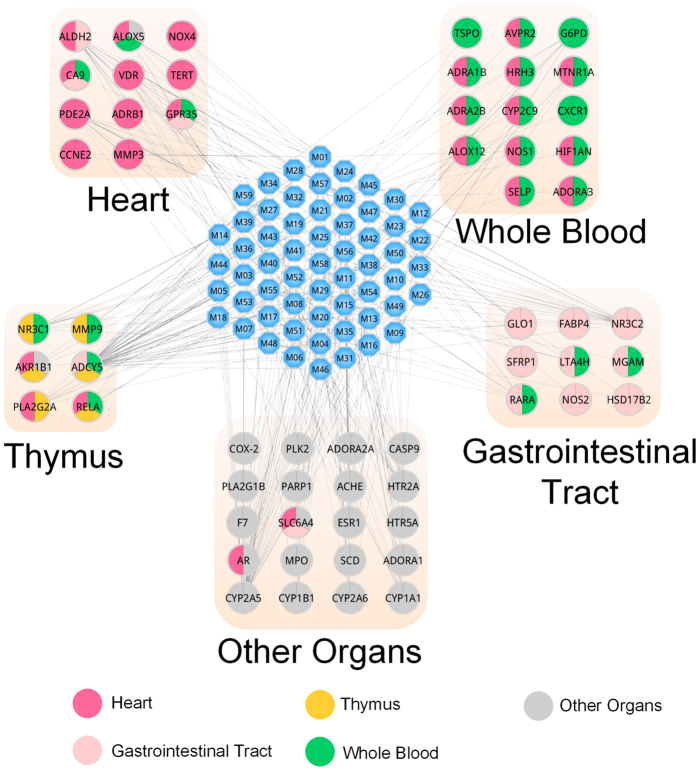
Target organ location map. The blue node represents the molecule and colored circles represents the target nodes along with the organs in which the target is located.

Understanding, on a system level, how the multi-organs respond to indications may facilitate the development of enhanced detection and treatment modalities for complex disease. By microarray analyses of mRNA expression, we found that 70 targets were mapped on 84 organs at different levels according to the BioGPS database (accessible at http://biogps.org)^115^. More importantly, we compared the expression patterns across different tissues, the tissue distribution network of the 70 targets are shown in Fig. 7. Based on the target expression pattern, the network is divided into several tissue modules, such as heart, stomach, whole blood and so on. Specifically, 24 targets contain higher mRNA expression in stomach than the average value of 84 tissues for each target. Therefore, these 24 stomach high-abundant targets are considered as therapeutic targets for GIDs, accounting for 34% of all the targets. There are 26 targets (accounting for 37% of all the targets) located in the heart, they are potential effective targets for the treatment of CVDs. Besides, most targets acted on two or more tissues, which suggests that these tissues are closely correlated. Consistent with this, patients with thymus hypoplasia have a high incidence of congenital heart disease^116^. Notably, the targets in whole blood are linked with tissues in almost all the forms. These results indicated that whole blood acts as the bridge and these tissues are closely related to the cardiovascular-gastrointestinal diseases.

#### Experimental Validation

**Figure 8:**
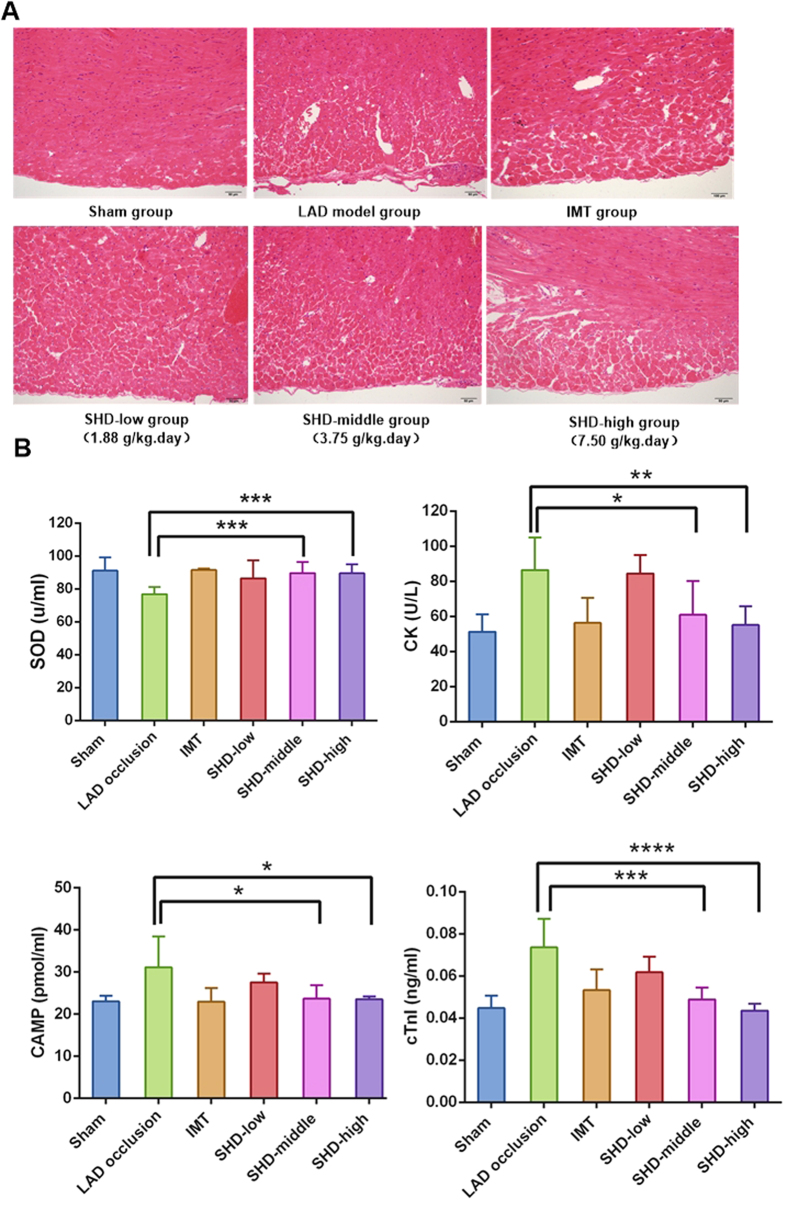
Effect of Sanhe Decoction (SHD) on myocardial ischemia pathological changes (**A**) and biochemical changes of SOD, CK, cAMP and cTnI serum levels (**B**) in left anterior descending coronary artery (LAD) occlusion rats. LAD occlusion rats were intragastricly treated with SHD-low 1.88 g/(kg.day), SHD-middle 3.75 g/(kg.day), SHD-high 7.5 g/(kg.day) and isosorbide mononitrate (IMT, 4 mg/kg) for one week. The myocardial pathological changes were analyzed by optical microscope analysis on the eighth day. The muscular fibers (A) (magnification, × 30,000) of myocardial ultrastructure images were taken. Values represent means ± SEM, n = 8. *P < 0.05, **P < 0.01, ***P < 0.001, ****P < 0.0001 vs. model.

To investigate whether SHD has a pathological effect on CVDs, we evaluated the effects of SHD on a rat model of left anterior descending coronary artery (LAD) occlusion. The light microscopy results showed that unimpaired integrity and intact layers of cells in hematoxylin-eosin (HE)-stained heart of non-LAD occlusion rats. In addition, there was no fibrous hyperplasia or edema in intercellular substance (Fig. 8(A)). In contrast, the LAD occlusion model group rats showed varying degrees of apomorphosis and necrosis of cardiac muscle cells. In addition, intercellular substance was slightly edema, fibrous hyperplasia and slight inflammatory cell invasion. Compared with LAD occlusion model group, pathological changes decreased significantly with less edema, fibrous hyperplasia and slight inflammatory cell invasion in rats pre-treated with SHD (SHD-low 1.88 g/(kg/day), SHD-middle 3.75 g/(kg/day) and SHD-high 7.5 g/(kg/day)) and isosorbide mononitrate. Notably, SHD-middle (3.75 g/(kg/day) group achieved the best myocardial protection effect, which significantly attenuated pathological changes (i.e., slight apomorphosis and necrosis, intercellular substance showed slight edema, and there was slight inflammatory cell invasion), which was similar as the positive group with isosorbide mononitrate (IMT). The results indicated that the myocardial damage was reduced by SHD from the morphological change.

Because SOD, CK, cAMP and serum cardiac troponin I (cTnI) concentrations are widely used as a biomarker for the detection of myocardial infarction, therefore, we measured SOD, CK, cAMP and cTnI in the serum to qualitatively assess the effect of SHD on the cardiac protection. As shown in Fig. 8(B), compared with model rats, SHD significantly increased the activity of antioxidant enzymes SOD and decreased levels of serum CK, cAMP and cTnI. These findings demonstrated that SHD treatment could effectively inhibit the production of free radicals of heart, and the results are consistent with those obtained by target protein function analysis.

## Discussion and Conclusion

It has been observed that CVDs have an etiologic relationship with GIDs^1^,^2^, and currently there is no effective treatment strategy for treating CVD and GID simultaneously in western medicine. Compared with western medicine, TCM has been used in the synchronic treatment of heart and stomach for a long time in China. However, the complex action mechanisms of TCM have hindered the development of effective therapy for such kind of systematic diseases. How to understand the TCM as the whole and identification of the shared potential active compounds, targets and biological processes become the bottleneck restrictions for modern TCM study.

(1) In this study, we first proposed a novel strategy integrating gene co-expression, meridian theory and systems pharmacology approach to explore the mechanisms of TCM in the synchronic treatment of CVDs and GIDs. Our main findings are as follows:

(2) A novel system is constructed to investigate the closeness analysis between genes of CVDs and GIDs. The shared genes of CVDs and GIDs were identified and the association was evaluated.

(3) Based on the meridian theory, herbs belonged to the heart meridian and stomach meridian were collected. These two sets of herbs were found to exhibit significant correlations through the Fisher’s test. More importantly, the combinations of HM and SM drugs were widely applied in clinical use of CPM for the treatment of CVDs and GIDs simultaneously.

The systems pharmacology approach, which integrates ADME evaluation, multi-target/pathway regulations, and multi-organ cooperation, was then applied to SHD to clarify the mechanisms involved in the co-treatment of heart and stomach diseases. In addition, an experiment was carried out to validate the therapeutic effects of SHD in CVDs.

In summary, this study provided an integrative analysis of related complex diseases by the systems pharmacology approach to find potential active compounds and understand the action mechanisms of TCM. Despite these potentially interesting findings above, further interpretation such as the drug-drug interaction and the herb dose-effect relationship is necessary to consider relied on experimental data analysis. Moreover, further experimental testing of these compound-target binding actions and molecular mechanism of active compounds *in vivo* will be required to support further assessments of potential clinical application.

## Materials and Methods

To understand the integrated treatment for co-occurring cardiovascular and gastrointestinal disorders, we firstly collected and analyzed the correlation of genes associated with CVDs and GIDs. Then, herbs of heart meridian (HM) and stomach meridian (SM) were extracted and their correlation was evaluated by Fisher’s test. In addition, these herbs were mapped into Chinese Patent Medicine (CPM) to investigate their clinical application and explore the principles of drug combination of heart and stomach meridians. Based on these systematic analyses of Chinese medicine, we selected Sanhe Decoction as a typical example to elaborate the molecular mechanisms of the co-treatment for cardiovascular and gastrointestinal disorders.

### The gene analysis of CVDs and GIDs

The genes associated with CVDs and GIDs were obtained from literature mining and several disease-gene databases: Therapeutic Target Database (TTD, http://bidd.nus.edu.sg/group/ttd/)^117^, DrugBank (http://www.drugbank.ca/)^118^, HIT (Herbal Ingredients,Targets Database, http://lifecenter.sgst.cn/hit/)^119^, PharmGKB (http://www.pharmgkb.org)^120^ and Comparative Toxicogenomics Database (CTD, http://ctdbase.org/)^121^.

To evaluate the correlation between genes associated with CVDs and GIDs, we mapped these genes to Human Protein-Protein Interaction (PPI) Network (from Hint Database: http://hint.yulab.org/)^122^. In the PPI network, the correlation between target a_i_ associated with CVDs and b_j_ associated with GIDs was calculated by the following formula:


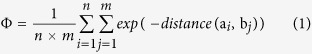


where n and m represent numbers of genes associated with CVDs and GIDs in the PPI network, respectively. Distance (a_i_, b_j_) represents the shortest path between gene a_i_ and b_j_ in the PPI network. If distance (a_i_, b_j_) is considered be infinite, it means that there is no association between CVDs’ and GIDs’ genes. The parameter exp (−distance (a_i_, b_j_)) could transfer the distance of two genes of CVDs and GIDs into the closeness between them. To further compare the associations between genes of CVDs and GIDs with that in the random two genes, we randomly selected two genes from PPI network and calculate the association between them, the process was repeated 1000 times.

### Analysis of HM and SM herbs and their application in Chinese Patent Medicine

In this work, the available information of the heart meridian (HM) and stomach meridian (SM) herbs were extracted from pharmacopoeia of the People’s republic of China (2010)^123^, which consists of 586 different species of drugs in text, such as herbs, plant oils and extracts. The Fisher’s test was applied to assess the relationship between HM and SM drugs.

To further investigate the clinical application of these HM and SM drugs, we restricted these drugs into a comprehensive encyclopedia of CPM, the National Chinese patent medicine^124^. CPM used in current clinical practice for CVDs (including angina, cardiodynia and angina pectoris,) and GIDs (such as gastrointestinal ulcers, chronic atrophic gastritis and chronic gastritis) were extracted for further study. Considering the fact that herbs related to heart and stomach meridians occurred in CPM for the treatment of CVDs and GIDs, *t*-test was used to evaluate the combination of HM and SM in the integrated treatment for co-occurring disorders.

### Systematic analysis of Sanhe Decoction

Here, Sanhe Decoction (consists of *Lilli Bulbus, Salvia Miltiorrhiza, Alpinia Officinarum Rhizoma, Amomum Villosum, Santail Albi Lignum, Lindera Aggregata* and *Cyperus Rotundus*) was selected as a case study, which is a famous Chinese medicine prescription in the treatment of epigastric pain including chronic atrophic gastritis, gastric ulcer and gastroesophageal reflux disease^125^ designed by TCM expert Jiao Shude. Besides, several previous studies have reported that SHD could be used to treat heart diseases as well^126^,^127^, but there was no study on the dissection of SHD for CVDs and GIDs. Therefore, we investigated the mechanisms of Sanhe Decoction in the treatment of CVDs and GIDs from a molecular to system level.

#### (1) ADME Screening

To screen for potentpharmaceutical compounds from Sanhe Decoction, an *in silico* ADME-systems evaluation model, which integrated drug-likeness (DL), oral bioavailability (OB), aqueous solubility (logS, the logarithm of aqueous solubility), lipophilicity (logP, logarithm of octanol-water partition coefficient) and Caco-2 permeability was proposed.

Lipophilicity: The lipophilicity was expressed as the partition coefficient P (log P), which is calculated by ALOGPS 2.1 software^128^. The value of log P less than 5 was selected for further analysis.

Aqueous solubility: Log S, a measure of aqueous solubility, which has been considered as an important factor in drug absorption and distribution. The value of Log S is also calculated by ALOGPS 2.1 software^128^ and the threshold value is range from −5 to −1.

Drug-likeness: To filter out the drug-like molecules, we have developed a database-dependent model to discriminate between drug-like and nondrug-like chemicals using the Tanimoto coefficient^129^. This model is constructed based on the molecular descriptors and Tanimoto coefficient (as displayed in Equation2).


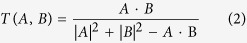


where A is the molecular properties of herbal ingredients, and B represents the average molecular properties of molecules in DrugBank database (http://www.drugbank.ca/) based on Dragon soft descriptors^130^. In this work, the molecules with satisfying drug-likeness index (DL ≥ 0.18) (average value for Drugbank) were selected as candidate compounds.

Oral bioavailability: OB, represents the rate and extent to which the active ingredient or active moiety is absorbed from a drug product and becomes available at the site of action^131^. Here the OB screening was performed by an in-house system OBioavail1.1^31^ and the compounds with OB ≥ 30% were kept in the database.

Caco-2 permeability: For an orally administered drug, the majority of drug absorption occurs in the small intestine where the presence of villi and microvilli greatly increases the surface available for absorption^132^. Here, we employed a robust *in silico* Caco-2 permeability prediction model PreCaco2^133^ to predict the drug absorption. We set the threshold of Caco-2 permeability to −0.4, because the compounds with Caco-2 value less than −0.4 are considered to be not permeable.

#### (2) Drug targeting

The identification of drug targets is a critical step for both the pharmaceutical industry and academic biomedical research. In this work, a newly developed weighted ensemble similarity (WES) method based on over 900,000 drug-target relations was proposed to predict the direct targets of drugs. The proposed method consists of two steps: (1) evaluating the significance of ligand structure parameters for each target and (2) calculating the potential unity of a molecule for a specific target based on the top-important chemical features that are strongly related to pharmacological properties, then adopting a statistical model to control for random similarity. The method integrates chemical, genomic and pharmacological data to build a high level model for acquiring higher prediction ability. More importantly, the reliability of the theoretical model is validated by a rat experiment. The WES model shows impressive performance for both internal and external validation data^30^.

#### (3) Predicting the mode of action of drugs

To characterize the interactions of drugs and target proteins, an *in silico* PreAM (Prediction of the Action Mode) model based on random forests (RF) algorithm was built. Firstly, drug structures and protein sequences were converted into numerical descriptors. Secondly, the multiple Compound-Target Interactions (CTIs) were represented by concatenating these chemical and protein descriptors, and minimal-redundancy–maximal-relevance (mRMR) was applied as a variable selected strategy to identify the best combination of descriptors to ensure the model with the highest predictive power. Thirdly, a random forests (RF) algorithm was trained to generate a nonlinear classifier tailored to CTIs with known action modes. The PreAM model shows impressive performance of prediction for drug-target interactions, with an overall accuracy of 97.3%, an activated prediction accuracy of 87.7%, an inhibited prediction accuracy of 99.8%^134^.

#### (4) Network construction

To further explore the multi-scale action mechanisms of herbal medicines in the prevention and treatment of CVDs and GIDs, we constructed two networks: Compound-Target network (C-T network) and Target-Pathway network (T-P network). In the network, the nodes represent compounds/targets/pathways, and edges represent they are linked with each other. The canonical pathways were extracted from KEGG database (http://www.genome.jp/kegg/)^92^. The enriched KEGG pathways of targets with a false discovery rate less than 0.05 by Fisher’s Exact test in DAVID database (https://david.ncifcrf.gov/)^96^ were analyzed^96^. Finally, the pathways were divided into several modules after the enrichment analysis. In these networks, degree (DD)^135^ is used to characterize the connectedness of a node. The degree of a node is the number of edges associated with it. The topological properties of these networks were analyzed using the Network Analysis plugin and CentiScaPe 1.2 of Cytoscape^136^.

#### (5) Compound organ location

To further explore the underlying mechanisms of Sanhe Decoction that provides therapeutic effects in CVDs and GIDs, it is essential to validate the functional and tissue expression profile of the protein targets at the organ level. We enriched the overrepresented gene ontology (GO) terms and checked the tissue distribution of the obtained targets. For GO analysis, the biological process of GO vocabulary (GOBP) was identified through GOBP terms by DAVID database (https://david.ncifcrf.gov/)^96^ and GOBP terms with adjusted P-values < 0.005 were observed. The target tissue distribution was determined based on the microarray analyses data of different tissue types lodged in the BioGPS bank (accessible at http://biogps.org)^115^.

Animal experimental validation: A total of 72 Sprague Dawley male rats (150–200 g) were obtained from Shanghai Super-B&K laboratory animal Co.Ltd. (Shanghai, China). All rats were housed in a controlled environment (temperature 23° ± 2 °C, relative humidity 50% ± 10%, 12 h light/dark cycle). SD rats were randomly divided into 6 groups with the equal number (n = 10): Sham-operated control group (Sham), LAD occlusion model control group(Vehicle), LAD occlusion rats treated with 1.88 g/(kg.day) SHD (SHD-low), 3.75 g/(kg.day) SHD (SHD-middle), 7.5 g/(kg.day) SHD (SHD-high), and 5.4 mg/kg isosorbide mononitrate (IMT). IMT is used as the positive control, which inhibits angiogenesis and mediates vasoconstriction to treat myocardial ischemia^137^.

The components of SHD were as follows: *Radix Salvia* 30 g, *Lilli Bulbus* 30 g, *Radix Linderae* 12 g, *Alpiniae Officinarum Rhizome* 9 g, *Santail Albi Lignum* 9 g, *Rhizoma Cyperi* 9 g and *Fructus Amomi* 6 g. All these herbal drugs were identified and prepared in fluid extract for use. For *in vivo* experimental validation, the route of SHD and IMT delivery were oral administration when the weight of rats was 300–350 g. Pre-treatment was given daily for a period of 7 days. Then, LAD occlusion rat model was used to assess the cardiac protection function of SHD and IMT. Following a procedure established in related reports^138^,^139^. Sham group underwent a thoracotomy without infarct induction. At 4 hours after LAD occlusion, rats were exposed to anesthesia. Prior to histopathologic examination, we harvested blood from the abdominal aorta and stored them in −20 °C temperature.

Animal experiments were conducted in accordance with the Guiding Principles for the Care and Use of Laboratory Animals. Our experimental protocol was approved by the Ethics Committee for Animal Experiments at State Key Laboratory of New-tech for Chinese Medicine Pharmaceutical Process of Jiangsu Kanion Parmaceutical Co.LtD. All efforts were made to minimize the number of the animals used and their suffering.

Histology lesion analysis: After taking blood samples from the abdominal aorta, we quickly removed the hearts of rats, storing them in 4% paraformaldehyde for histological analysis. Next, we cut consecutive cross-sections (4 μm thick) of the hearts, and stained with hematoxylin-eosin (HE). Morphology were determined under an Olympus BH20 microscope (Olympus; Tokyo, Japan).

Biochemical analysis: We collected blood samples and measured serum levels of superoxide dismutase (SOD), creatine kinase (CK), cyclic adenosine monophosphate (cAMP), and cardiac troponin I (cTnI) using chemical colorimetry assay kits (Nanjing Jiancheng Bioengineering Institute) according to manufacturer instructions.

Statistical analysis: Mean values ± S.E. was calculated from independent experiments. Data were analyzed by Student’s t test and one-way analysis of variance. *P* value less than 0.05 was considered significant.

## Additional Information

**How to cite this article**: Zhang, W. *et al*. Systems Pharmacology Dissection of the Integrated Treatment for Cardiovascular and Gastrointestinal Disorders by Traditional Chinese Medicine. *Sci. Rep.* **6**, 32400; doi: 10.1038/srep32400 (2016).

## Supplementary Material

Supplementary Information

## Figures and Tables

**Figure 1:**
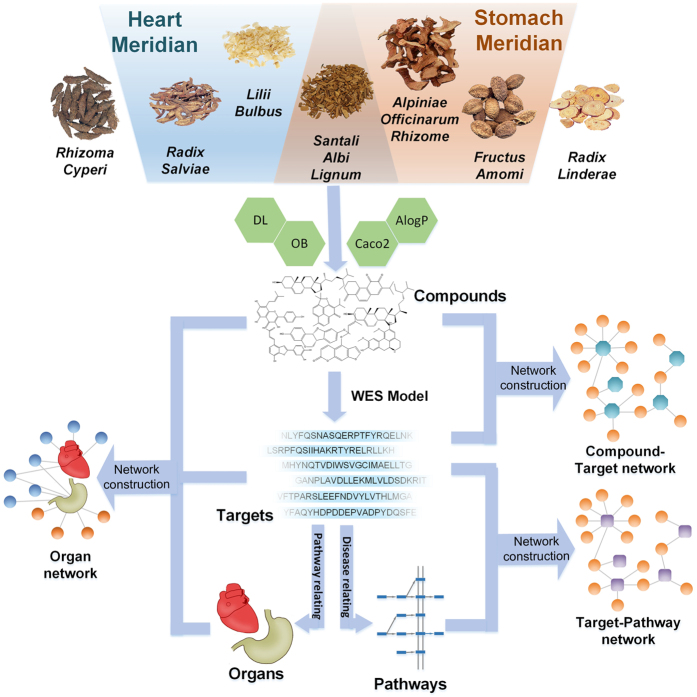
Systems pharmacology approach framework.

**Table 1:** The shared genes of CVDs and GIDs.

GeneSymbol	Gene name	Types of CVDs	Types of GIDs
ABCB1	ATP-binding cassette, sub-family B (MDR/TAP), member 1	Heart Defects, Congenital	Gastroesophageal Reflux
ACE	angiotensin I converting enzyme	Heart Failure, Cardiovascular Diseases	Stomach Neoplasms
ADRB1	adrenoceptor beta 1	Heart Failure, Cardiovascular Diseases	Stomach Neoplasms
ADRB2	adrenoceptor beta 2, surface	Heart Failure	Stomach Neoplasms
AHR	aryl hydrocarbon receptor	Heart Defects, Congenital; Heart diseases	Stomach Neoplasms
ALB	albumin	Heart Diseases; Heart Failure; Cardiovascular Diseases	Stomach Neoplasms
APEX1	APEX nuclease (multifunctional DNA repair enzyme) 1	Heart Diseases	Stomach Neoplasms
AVP	arginine vasopressin	Heart Failure	Gastrointestinal Hemorrhage
CAT	catalase	Heart Failure	Stomach Ulcer
CSF2	colony stimulating factor 2 (granulocyte-macrophage)	Heart Failure	Gastrointestinal Hemorrhage
CSF3	colony stimulating factor 3 (granulocyte)	Heart Failure; Heart Diseases	Gastrointestinal Diseases
CYP2C19	cytochrome P450, family 2, subfamily C, polypeptide 19	Cardiovascular Diseases	Gastroesophageal Reflux
EDN1	endothelin 1	Heart Failure; Cardiovascular Diseases; Heart Defects, Congenital; Cardiovascular Abnormalities; Cardiovascular disease, unspecified; Ischemic heart disease	Gastrointestinal Diseases
F2R	coagulation factor II (thrombin) receptor	Cardiovascular Disorders	Stomach Neoplasms
GCG	glucagon	Heart Failure; Heart Diseases	Stomach Diseases
GDF15	growth differentiation factor 15	Heart Failure; Cardiovascular disease, unspecified	Gastrointestinal Neoplasms
GHRL	ghrelin/obestatin prepropeptide	Heart Failure	Stomach Ulcer
GHSR		Cardiovascular disease, unspecified	Gastrointestinal Diseases and Disorders, miscellaneous
HMOX1	heme oxygenase (decycling) 1	Heart Failure; Cardiovascular disease, unspecified	Stomach Neoplasms; Gastroparesis
HSPB1	heat shock 27 kDa protein 1	Heart Failure	Stomach Neoplasms
IL1B	interleukin 1, beta	Heart Failure; Heart Valve Diseases	Stomach Ulcer; Stomach Neoplasms; Gastritis, Atrophic
IL6	interleukin 6	Heart Failure	Stomach Neoplasms
MT2A	metallothionein 2A	Heart Diseases	Stomach Neoplasms
MTHFR	methylenetetrahydrofolate reductase (NAD(P)H)	Cardiovascular Diseases; Heart Defects, Congenital	Stomach Neoplasms;
NOS2	nitric oxide synthase 2, inducible	Heart Failure, Cardiovascular Abnormalities	Stomach Ulcer
NOS3	nitric oxide synthase 3 (endothelial cell)	Cardiovascular Diseases; Heart Failure	Stomach Ulcer, Stomach Neoplasms
NRG1	neuregulin 1	Heart Failure	Stomach Ulcer
PLAU	plasminogen activator, urokinase	Heart Rupture, Post-Infarction	Stomach Neoplasms
POMC	proopiomelanocortin	Heart Failure	Gastrointestinal Diseases
PPARG	peroxisome proliferator-activated receptor gamma	Ischemic heart disease	Stomach Neoplasms
PTGS1	prostaglandin-endoperoxide synthase 1 (prostaglandin G/H synthase and cyclooxygenase)	Heart Failure	Stomach Ulcer
PTGS2	prostaglandin-endoperoxide synthase 2 (prostaglandin G/H synthase and cyclooxygenase)	Cardiovascular Diseases; Heart Failure	Stomach Ulcer; Stomach Neoplasms
PYCARD	PYD and CARD domain containing	Heart Valve Diseases	Stomach Neoplasms
RELA		Cardiovascular disease, unspecified	Gastric cancer
SERPINE1	serpin peptidase inhibitor, clade E (nexin, plasminogen activator inhibitor type 1), member 1	Heart Failure	Stomach Neoplasms
SOD2	superoxide dismutase 2, mitochondrial	Heart Failure	Stomach Neoplasms
TNF	tumor necrosis factor	Congestive heart failure	Stomach Ulcer; Stomach Neoplasms
VEGFA	vascular endothelial growth factor A	Heart Diseases; Heart Failure	Stomach Ulcer
ACHE	Acetylcholinesterase	Heart Failure	Stomach Tumor
AR	Androgen Receptor	Atherosclerosis	Gastric Cancer
CYP2A6	Cytochrome P450 2A6	Cardiovascular Disease	Gastric Carcinoma
ESR1	Estrogen Receptor	Ischemia Reperfusion Injury	Gastric Cancer
GLO1	Glyoxalase I	Diabetic Cardiomyopathy	Gastric Cancer
MMP9	Matrix metalloproteinase 9	Heart Failure	Gastric Carcinoma
NR3C1	Glucocorticoid receptor	Coronary Heart Disease	Gastric Cancer
PLA2G2A	Phospholipase A2 group IIA	Atherosclerosis	Gastric Cancer
PLK2	Polo-Like Kinase 2	Myocardial Infarction	Stomach Tumor

**Table 2:** The shared herbs that belong to heart and stomach meridians.

Herb names	Pinyin	Meridians	
*Acori Tatarinowii Rhizoma*	Shichangpu	heart, stomach	pungent, bitter, temperature
*Allii Macrostemonis Bulbus*	Xiebai	heart, lung, stomach, large intestine	pungent, bitter, temperature
*Ampelopsis Radix*	Bailian	heart, stomach	bitter, little cold
*Bambusae Caulis in Taenias*	Zhuru	lung, stomach, heart, gallbladder	sweet, little cold
*Baphicacanthis Cusiae Rhizoma et Radix*	Nanbanlangen	heart, stomach	bitter, cold
*Coptidis Rhizoma*	Huanglian	heart, spleen, stomach, liver, gallbladder, large intestine	bitter, cold
*Glycyrrhizae Radix et Rhizoma Praeparata Cum Melle*	Zhigancao	heart, lung, spleen, stomach	sweet, natured
*Hippophae Fructus*	Shaji	spleen, stomach, lung, heart	sour, astringent, temperature
*Hyoscyami Semen*	Tianxianzi	heart, stomach, liver	bitter, pungent, temperature, large toxic
*Isatidis Folium*	Daqingye	heart, stomach	bitter, cold
*Isatidis Radix*	Banlangen	heart, stomach	bitter, cold
*Liriopes Radix*	Shanmaidong	heart, lung, stomach	sweet, little bitter, little cold
*Lonicerae Flos*	Shanyinhua	lung, heart, stomach	sweet, cold
*Lonicerae Japonicae Flos*	Jinyinhua	lung, heart, stomach	sweet, cold
*Lophatheri Herba*	Danzhuye	heart, stomach, small intestine	sweet, bland, cold
*Ophiopogonis Radix*	Maidong	heart, lung, stomach	sweet, little bitter, little cold
*Polygoni Tinctorii Folium*	Liaodaqingye	heart, stomach	bitter, cold
*Santali Albi Lignum*	Tanxiang	spleen, stomach, heart, lung	pungent, temperature
*Sophorae Flavescentis Radix*	Kushen	heart, liver, stomach, large intestine, bladder	bitter, cold
*Tamaricis Cacumen*	Xiheliu	heart, lung, stomach	sweet, pungent, natured
*Zingiberis Rhizoma*	Ganjiang	spleen, stomach, kidney, heart, lung	pungent, hot

**Table 3:**
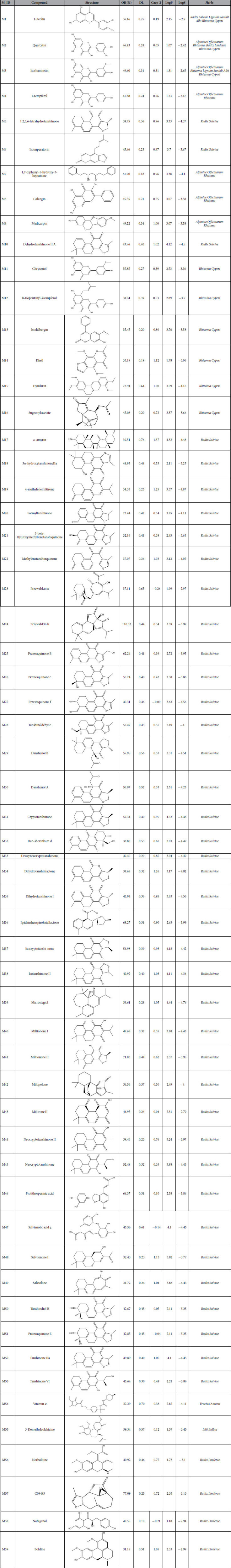
Active constituents of herbs in Sanhe Decoration and their corresponding ADME parameters.

**Table 4:** The GIDs target information.

Gene name	Protein name	Degree
ESR1	Estrogen Receptor	18
NR3C1	Glucocorticoid receptor	12
AR	Androgen Receptor	8
PlA2G2A	Phospholipase A2 group IIA	8
CYP2A6	Cytochrome P450 2A6	7
CYP1B1	Cytochrome P450 1B1	6
GLO1	Glyoxalase I	4
ACHE	Acetylcholinesterase	3
NOS1	Nitric Oxide Synthase, brain	3
HSD17B2	Estradiol 17-beta-dehydrogenase 2	2
PLK2	Polo-Like Kinase 2	2
CCNE2	Cyclin-dependent kinase 2	2
MMP9	Matrix metalloproteinase 9	2
CYP2C9	Cytochrome P450 2C9	2
SCD	Acyl-CoA desaturase	1
COX-2	Cyclooxygenase-2	1
HRH3	Histamine H3 Receptor	1
MGAM	Maltase-glucoamylase	1
SELP	P-selectin	1
HIF1AN	Hypoxia-inducible factor 1-alpha inhibitor	1
CA9	Carbonic anhydrase 9	1
CASP9	Caspase-9	1
ADRA1B	Alpha-1b adrenergic receptor	1
TERT	Telomerase reverse transcriptase	1

**Table 5:** The CVDs target information.

Gene_Name	Protein Name	Degree
ADCY5	Adenylate cyclase type V	47
CYP2A5	Cytochrome P450 2A5	35
NR3C2	Glucocorticoid receptor	20
HTR2A	5-hydroxytryptamine receptor 2A	18
ESR1	Estrogen Receptor	18
ALDH2	Aldehyde dehydrogenase	15
CYP1A1	Cytochrome P450 1A1	13
NR3C1	Glucocorticoid receptor	12
PDE2A	Phosphodiesterase 2A	12
VDR	Vitamin D receptor	9
AKR1B1	Aldose reductase	9
AR	Androgen Receptor	8
ALOX5	5-Lipoxygenase	8
G6PD	glucose-6-phosphate 1-dehydrogenase isoform b	8
PLA2G2A	Phospholipase A2 group IIA	8
RARA	Retinoic acid receptor alpha	8
CYP2A6	Cytochrome P450 2A6	7
MTNR1A	Melatonin receptor 1A	5
ALOX12	Arachidonate 12-lipoxygenase	4
GLO1	Glyoxalase I	4
ACHE	Acetylcholinesterase	3
HTR5A	Serotonin 5a receptor	3
LTA4H	Leukotriene A4 Hydrolase	3
SLC6A4	Serotonin Transporter	3
RELA	v-rel reticuloendotheliosis viral oncogene homolog A isoform 1	3
ADORA1	Adenosine A1 receptor	3
ALOX5	5-Lipoxygenase	2
PLK2	Polo-Like Kinase 2	2
TSPO	Translocator protein	2
NOX4	NADPH oxidase 4	2
GPR35	G protein-coupled receptor 35	2
ADORA2A	Adenosine Receptor A2A	2
MMP9	Matrix metalloproteinase 9	2
ADORA3	Adenosine receptor A3	2
ADRA2B	Alpha-2b adrenergic receptor	2
MMP3	Matrix metalloproteinase-3	2
FABP4	Fatty acid binding protein adipocyte	1
SFRP1	Secreted frizzled-related protein 1	1
PLA2G1B	Phospholipase A2	1
AVPR2	Vasopressin V2 Receptor	1
CXCR1	Interleukin-8 receptor A	1
MPO	Myeloperoxidase	1
SORD	Sorbitol dehydrogenase	1
F7	Coagulation factor III/VII	1
PARP1	Poly [ADP-ribose] polymerase-1	1
ADRB1	Beta-1 adrenergic receptor	1
