# Supplementary material for: Systems Pharmacology Dissection of the Integrated Treatment for Cardiovascular and Gastrointestinal Disorders by Traditional Chinese Medicine
Source: Sci Rep. 2016 Sep 6;6:32400. doi: 10.1038/srep32400 (PMC5011655; doi:10.1038/srep32400)
Supplement: Supplementary Information [file srep32400-s1.pdf]

Systems-pharmacology dissection of the Integrated Treatment for Cardiovascular and Gastrointestinal Disorders by Traditional Chinese Medicine

Wenjuan Zhang[1,2], Qin Tao[1,2], Zihu Guo[1,2], Yingxue Fu[1,2], Xuetong Chen[1,2], Piar Ali Shar[1,2], Mohamed Shahen[1,2], Jinglin Zhu[3], Jun Xue[3], Yaofei Bai[1,2], Ziyin Wu[1,2], Zhenzhong Wang[4], Wei Xiao[4*], Yonghua Wang[1,2*]

Supplementary S1 Gene information associated with CVDs

Disease_name	Gene_sympol	Gene_name
Heart Failure	PTI	pancreatic trypsin inhibitor
Heart Septal Defects, Atrial	PQBP1	polyglutamine binding protein 1
Heart Defects, Congenital	IRX5	iroquois homeobox 5
Heart Septal Defects	CITED2	Cbp/p300-interacting transactivator, with Glu/Asp-rich carboxy-terminal domain, 2
Heart Failure	FBLN5	fibulin 5
Cardiovascular Diseases	CETP	cholesteryl ester transfer protein, plasma
Heart Failure	PPARGC1A	peroxisome proliferator-activated receptor gamma, coactivator 1 alpha
Heart Valve Diseases	RIPK3	receptor-interacting serine-threonine kinase 3
Heart Diseases	ATP5K	ATP synthase, H+ transporting, mitochondrial F1F0 complex, subunit E
Heart Diseases	ABCC2	ATP-binding cassette, sub-family C (CFTR/MRP), member 2
Heart Diseases	HSPB6	heat shock protein, alpha-crystallin-related, B6
Heart Valve Diseases	COL1A1	collagen, type I, alpha 1
Heart Valve Diseases	COL1A2	collagen, type I, alpha 2
Conotruncal Heart Malformations	NKX2-6	NK2 homeobox 6
Cardiovascular Diseases	CRP	C-reactive protein, pentraxin-related
Heart Diseases	CRP	C-reactive protein, pentraxin-related
Heart Failure	CRP	C-reactive protein, pentraxin-related
Heart Failure	CS	citrate synthase
Heart Failure	CSF2	colony stimulating factor 2 (granulocyte-macrophage)
Heart Failure	CSF3	colony stimulating factor 3 (granulocyte)
Heart Diseases	CSF3	colony stimulating factor 3 (granulocyte)
Heart Diseases	UNC45B	unc-45 homolog B (C. elegans)

Cardiovascular Diseases	CST3	cystatin C
Heart Failure	CTGF	connective tissue growth factor
Heart Diseases	ADRA2A	adrenoceptor alpha 2A
Heart Failure	ADRA2C	adrenoceptor alpha 2C
Heart Failure	ADRB1	adrenoceptor beta 1
Cardiovascular Diseases	ADRB1	adrenoceptor beta 1
Heart Diseases	CYBA	cytochrome b-245, alpha polypeptide
Heart Failure	CYBB	cytochrome b-245, beta polypeptide
Heart Failure	ADRB3	adrenoceptor beta 3
Cardiovascular Diseases	CYP2C19	cytochrome P450, family 2, subfamily C, polypeptide 19
Heart Failure	ACE	angiotensin I converting enzyme
Cardiovascular Diseases	ACE	angiotensin I converting enzyme
Heart Defects, Congenital	JAG1	jagged 1
Heart Defects, Congenital	RCAN1	regulator of calcineurin 1
Heart Diseases	AGT	angiotensinogen (serpin peptidase inhibitor, clade A, member 8)
Heart Failure	AGT	angiotensinogen (serpin peptidase inhibitor, clade A, member 8)
Cardiovascular Diseases	AGT	angiotensinogen (serpin peptidase inhibitor, clade A, member 8)
Heart Failure	AGTR1	angiotensin II receptor, type 1
Heart Diseases	ABCB1A	ATP-binding cassette, sub-family B (MDR/TAP), member 1A
Heart Defects, Congenital	ECE1	endothelin converting enzyme 1
Heart Failure	EDN1	endothelin 1
Cardiovascular Diseases	EDN1	endothelin 1
Heart Defects, Congenital	EDN1	endothelin 1
Cardiovascular Abnormalities	EDN1	endothelin 1
Heart Defects, Congenital	EDNRA	endothelin receptor type A
Heart Defects, Congenital	AHR	aryl hydrocarbon receptor
Heart Valve Diseases	CCL6	chemokine (C-C motif) ligand 6
Cardiovascular Abnormalities	ANKS6	ankyrin repeat and sterile alpha motif domain containing 6
Heart Failure	EPHX2	epoxide hydrolase 2, cytoplasmic
Heart Septal Defects, Ventricular	EPO	erythropoietin
Heart Diseases	EPO	erythropoietin
Heart Diseases	ALB	albumin
Cardiovascular Diseases	ALB	albumin
Heart Failure	ALB	albumin
Heart Defects, Congenital	EYA1	EYA transcriptional coactivator and phosphatase 1
Heart Injuries	ALDH2	aldehyde dehydrogenase 2 family (mitochondrial)
Heart Failure	TRP53	transformation related protein 53
Heart Valve Diseases	FCGR1A	Fc fragment of IgG, high affinity Ia, receptor (CD64)
Heart Failure	TUBB5	tubulin, beta 5 class I
Cardiovascular Abnormalities	FGF8	fibroblast growth factor 8 (androgen-induced)
Cardiovascular Abnormalities	FOXC2	forkhead box C2
Heart Diseases	SIRT1	sirtuin 1
Heart Defects, Congenital	FOLR1	folate receptor 1 (adult)
Heart Defects, Congenital	NIPBL	Nipped-B homolog (Drosophila)

Heart Failure	GATM	glycine amidinotransferase (L-arginine:glycine amidinotransferase)
Heart Failure	GCG	glucagon
Heart Diseases	GCG	glucagon
Conotruncal Heart Malformations	GDF1	growth differentiation factor 1
Heart Failure	MSTN	myostatin
Cardiovascular Diseases	GH1	growth hormone 1
Hypoplastic Left Heart Syndrome	GJA1	gap junction protein, alpha 1, 43kDa
Heart Failure	NOX1	NADPH oxidase 1
Heart Defects, Congenital	AFF4	AF4/FMR2 family, member 4
Heart Defects, Congenital	GNA11	guanine nucleotide binding protein (G protein), alpha 11 (Gq class)
Heart Defects, Congenital	GNAQ	guanine nucleotide binding protein (G protein), q polypeptide
Heart Defects, Congenital	GP1BB	glycoprotein Ib (platelet), beta polypeptide
Cardiovascular Diseases	GPX1	glutathione peroxidase 1
Heart Failure	GPX4	glutathione peroxidase 4
Heart Valve Diseases	PYCARD	PYD and CARD domain containing
Heart Failure	CXCL2	chemokine (C-X-C motif) ligand 2
Heart Failure	GSK3B	glycogen synthase kinase 3 beta
Heart Diseases	HADHB	hydroxyacyl-CoA dehydrogenase/3-ketoacyl-CoA thiolase/enoyl-CoA hydratase (trifunctional protein), beta subunit
Heart Diseases	AHR2	aryl hydrocarbon receptor 2
Heart Diseases	HDAC1	histone deacetylase 1
Heart Failure	NRG1	neuregulin 1
Heart Failure	HIF1A	hypoxia inducible factor 1, alpha subunit (basic helix-loop-helix transcription factor)
Heart Failure	HMOX1	heme oxygenase (decycling) 1
Heart Defects, Congenital	HOXA1	homeobox A1
Heart Defects, Congenital	HOXA3	homeobox A3
Cardiovascular Diseases	HP	haptoglobin
Heart Failure	APCS	amyloid P component, serum
Heart Diseases	APEX1	APEX nuclease (multifunctional DNA repair enzyme) 1
Heart Failure	HSPB1	heat shock 27kDa protein 1
Heart Valve Diseases	HTR2B	5-hydroxytryptamine (serotonin) receptor 2B, G protein-coupled
Heart Failure	HTR2B	5-hydroxytryptamine (serotonin) receptor 2B, G protein-coupled
Cardiovascular Diseases	APOB	apolipoprotein B
Cardiovascular Diseases	ICAM1	intercellular adhesion molecule 1
Cardiovascular Diseases	APOC3	apolipoprotein C-III
Heart Failure	IFNG	interferon, gamma
Heart Arrest	IFNG	interferon, gamma
Cardiovascular Diseases	APOE	apolipoprotein E
Heart Failure	IL1B	interleukin 1, beta
Heart Valve Diseases	IL1B	interleukin 1, beta
Heart Failure	IL6	interleukin 6
Heart Valve Diseases	IL18	interleukin 18
Heart Failure	INS	insulin

Heart Arrest	INS	insulin
Heart Failure	ITGB1	integrin, beta 1 (fibronectin receptor, beta polypeptide, antigen CD29 includes MDF2, MSK12)
Heart Valve Diseases	ITGB2	integrin, beta 2 (complement component 3 receptor 3 and 4 subunit)
Heart Valve Diseases	JAK2	Janus kinase 2
Heart Valve Diseases	LCN2	lipocalin 2
Heart Block	LMNA	lamin A/C
Heart-Hand Syndrome, Slovenian Type	LMNA	lamin A/C
Cardiovascular Diseases	LPA	lipoprotein, Lp(a)
Cardiovascular Diseases	LPL	lipoprotein lipase
Heart Valve Diseases	LSP1	lymphocyte-specific protein 1
Heart Diseases	MIR34A	microRNA 34a
Heart Failure	NR3C2	nuclear receptor subfamily 3, group C, member 2
Cardiovascular Diseases	MPO	myeloperoxidase
Heart Diseases	ABCC1	ATP-binding cassette, sub-family C (CFTR/MRP), member 1
Heart Diseases	MT2A	metallothionein 2A
Cardiovascular Diseases	MTHFR	methylenetetrahydrofolate reductase (NAD(P)H)
Heart Defects, Congenital	MTHFR	methylenetetrahydrofolate reductase (NAD(P)H)
Heart Septal Defects, Atrial	MYH6	myosin, heavy chain 6, cardiac muscle, alpha
Heart Injuries	MYL1	myosin, light chain 1, alkali; skeletal, fast
Heart Diseases	NCF4	neutrophil cytosolic factor 4, 40kDa
Heart Failure	ATP1A3	ATPase, Na+/K+ transporting, alpha 3 polypeptide
Heart Failure	NOS2	nitric oxide synthase 2, inducible
Cardiovascular Abnormalities	NOS2	nitric oxide synthase 2, inducible
Cardiovascular Diseases	NOS3	nitric oxide synthase 3 (endothelial cell)
Heart Failure	NOS3	nitric oxide synthase 3 (endothelial cell)
Heart Valve Diseases	NOTCH1	notch 1
Heart Failure	ACHE	acetylcholinesterase
Atherosclerosis	AR	androgen Receptor
Cardiovascular Disease	CYP2A6	cytochrome P450 2A6
Ischemia Reperfusion Injury	ESR1	estrogen receptor
Diabetic Cardiomyopathy	GLO1	glyoxalase I
Heart Failure	MMP9	matrix metalloproteinase 9
Coronary Heart Disease	NR3C1	glucocorticoid receptor
Atherosclerosis	PLA2G2A	phospholipase A2 group IIA
Myocardial Infarction	PLK2	polo-Like Kinase 2
Cardiovascular Diseases	NPY	neuropeptide Y
Heart Failure	ATP2A1	ATPase, Ca++ transporting, cardiac muscle, fast twitch 1
Heart Failure	NPPA	natriuretic peptide A
Heart Diseases	NPPB	natriuretic peptide B
Cardiovascular Diseases	NPPB	natriuretic peptide B
Heart Defects, Congenital	NPPB	natriuretic peptide B
Heart Failure	NPPB	natriuretic peptide B

Heart Diseases	ATP2A2	ATPase, Ca++ transporting, cardiac muscle, slow twitch 2
Heart Failure	ATP2A2	ATPase, Ca++ transporting, cardiac muscle, slow twitch 2
Heart Failure	NPR1	natriuretic peptide receptor 1

Supplementary S2 Gene information associated with GIDs

Disease_name	Gene_symbol	Gene_name
Stomach Neoplasms	CDH2	cadherin 2, type 1, N-cadherin (neuronal)
Stomach Neoplasms	TRAP1	TNF receptor-associated protein 1
Stomach Neoplasms	EBI3	epstein-Barr virus induced 3
Stomach Neoplasms	CDK4	cyclin-dependent kinase 4
Stomach Neoplasms	MSLN	mesothelin
Stomach Neoplasms	CDKN1A	cyclin-dependent kinase inhibitor 1A (p21, Cip1)
Stomach Neoplasms	CDKN1B	cyclin-dependent kinase inhibitor 1B (p27, Kip1)
Stomach Neoplasms	CDKN2A	cyclin-dependent kinase inhibitor 2A
Stomach Neoplasms	CDKN2D	cyclin-dependent kinase inhibitor 2D (p19, inhibits CDK4)
Stomach Neoplasms	CNPY2	canopy FGF signaling regulator 2
Stomach Neoplasms	FST	follistatin
Stomach Neoplasms	NOP56	NOP56 ribonucleoprotein
Stomach Neoplasms	ARL6IP5	ADP-ribosylation factor-like 6 interacting protein 5
Stomach Neoplasms	CCT7	chaperonin containing TCP1, subunit 7 (eta)
Stomach Neoplasms	CTSC	cathepsin C
Stomach Neoplasms	WIF1	WNT inhibitory factor 1
Stomach Neoplasms	CHEK2	checkpoint kinase 2
Stomach Neoplasms	CKB	creatine kinase, brain
Stomach Neoplasms	LRRC3B	leucine rich repeat containing 3B
Stomach Neoplasms	CLCN3	chloride channel, voltage-sensitive 3
Stomach Neoplasms	CLN3	ceroid-lipofuscinosis, neuronal 3
Stomach Neoplasms	PLIN2	perilipin 2
Stomach Neoplasms	MBD3L2	methyl-CpG binding domain protein 3-like 2
Stomach Neoplasms	DCBLD2	discoidin, CUB and LCCL domain containing 2
Stomach Neoplasms	KLF6	kruppel-like factor 6
Stomach Ulcer	ADM	adrenomedullin
Stomach Neoplasms	CLDN3	claudin 3
Gastrointestinal Hemorrhage	CSF2	colony stimulating factor 2 (granulocyte-macrophage)
Gastrointestinal Diseases	CSF3	colony stimulating factor 3 (granulocyte)
Stomach Neoplasms	CST1	cystatin SN
Stomach Neoplasms	CTNNA2	catenin (cadherin-associated protein), alpha 2
Stomach Neoplasms	CTSL	cathepsin L
Stomach Neoplasms	ADRB1	adrenoceptor beta 1
Stomach Neoplasms	ADRB2	adrenoceptor beta 2, surface
Stomach Neoplasms	CYP2A6	cytochrome P450, family 2, subfamily A, polypeptide 6
Stomach Neoplasms	ACE	angiotensin I converting enzyme
Stomach Neoplasms	DDB1	damage-specific DNA binding protein 1, 127kDa
Stomach Neoplasms	GADD45A	growth arrest and DNA-damage-inducible, alpha

Stomach Neoplasms	DES	desmin
Stomach Neoplasms	AFP	alpha-fetoprotein
Gastrointestinal Stromal Tumors	DMD	dystrophin
Stomach Neoplasms	DNMT1	DNA (cytosine-5-)-methyltransferase 1
Stomach Neoplasms	DNMT3B	DNA (cytosine-5-)-methyltransferase 3 beta
Stomach Neoplasms	DPAGT1	dolichyl-phosphate (UDP-N-acetylglucosamine) N-acetylglucosaminephosphotransferase 1 (GlcNAc-1-P transferase)
Gastroesophageal Reflux	ABAT	4-aminobutyrate aminotransferase
Stomach Neoplasms	DPYD	dihydropyrimidine dehydrogenase
Stomach Neoplasms	HBEGF	heparin-binding EGF-like growth factor
Stomach Neoplasms	AGTR2	angiotensin II receptor, type 2
Stomach Neoplasms	TYMP	thymidine phosphorylase
Stomach Neoplasms	ECHS1	enoyl CoA hydratase, short chain, 1, mitochondrial
Gastrointestinal Diseases	EDN1	endothelin 1
Stomach Neoplasms	EEF1A1	eukaryotic translation elongation factor 1 alpha 1
Stomach Neoplasms	EEF1A2	eukaryotic translation elongation factor 1 alpha 2
Stomach Ulcer	EGF	epidermal growth factor
Stomach Neoplasms	EGFR	epidermal growth factor receptor
Stomach Neoplasms	AHR	aryl hydrocarbon receptor
Stomach Neoplasms	REG1	regenerating islet-derived 1
Stomach Neoplasms	ENO1	enolase 1, (alpha)
Stomach Neoplasms	ERBB2	erb-b2 receptor tyrosine kinase 2
Gastrointestinal Neoplasms	ERBB2	erb-b2 receptor tyrosine kinase 2
Stomach Neoplasms	ERCC1	excision repair cross-complementation group 1
Stomach Neoplasms	ERCC2	excision repair cross-complementation group 2
Stomach Neoplasms	ALB	albumin
Stomach Neoplasms	F2R	coagulation factor II (thrombin) receptor
Gastroenteritis	F2RL1	coagulation factor II (thrombin) receptor-like 1
Stomach Neoplasms	ALDH1A3	aldehyde dehydrogenase 1 family, member A3
Stomach Neoplasms	FBP1	fructose-1,6-bisphosphatase 1
Stomach Neoplasms	FGFR2	fibroblast growth factor receptor 2
Stomach Neoplasms	FGG	fibrinogen gamma chain
Stomach Neoplasms	FHIT	fragile histidine triad
Stomach Neoplasms	FKBP2	FK506 binding protein 2, 13kDa
Stomach Neoplasms	ALDOB	aldolase B, fructose-bisphosphate
Stomach Neoplasms	FAM168A	family with sequence similarity 168, member A
Stomach Neoplasms	BOP1	block of proliferation 1
Stomach Neoplasms	ALOX5	arachidonate 5-lipoxygenase
Stomach Neoplasms	ATP6V0D2	ATPase, H+ transporting, lysosomal 38kDa, V0 subunit d2
Gastrointestinal Diseases	GAST	gastrin
Stomach Neoplasms	GAST	gastrin
Stomach Neoplasms	FYN	FYN proto-oncogene, Src family tyrosine kinase
Stomach Neoplasms	PRDX5	peroxiredoxin 5

Stomach Neoplasms	SERBP1	SERPINE1 mRNA binding protein 1
Stomach Neoplasms	ZBTB20	zinc finger and BTB domain containing 20
Stomach Diseases	GCG	glucagon
Stomach Neoplasms	GREM1	gremlin 1, DAN family BMP antagonist
Stomach Neoplasms	ACAD8	acyl-CoA dehydrogenase family, member 8
Stomach Neoplasms	SNX5	sorting nexin 5
Stomach Neoplasms	FILIP1	filamin A interacting protein 1
Stomach Neoplasms	PRPF19	pre-mRNA processing factor 19
Stomach Neoplasms	GLI3	GLI family zinc finger 3
Stomach Neoplasms	MRPS18B	mitochondrial ribosomal protein S18B
Stomach Neoplasms	MRPL13	mitochondrial ribosomal protein L13
Stomach Neoplasms	PYCARD	PYD and CARD domain containing
Stomach Neoplasms	GSTP1	glutathione S-transferase pi 1
Stomach Neoplasms	ABT1	activator of basal transcription 1
Stomach Neoplasms	CPSF1	cleavage and polyadenylation specific factor 1, 160kDa
Stomach Diseases	ANXA1	annexin A1
Stomach Neoplasms	ANXA5	annexin A5
Stomach Ulcer	NRG1	neuregulin 1
Gastrointestinal Neoplasms	HIC1	hypermethylated in cancer 1
Stomach Neoplasms	HMOX1	heme oxygenase (decycling) 1
Gastroparesis	HMOX1	heme oxygenase (decycling) 1
Stomach Neoplasms	HNRNPL	heterogeneous nuclear ribonucleoprotein L
Stomach Neoplasms	HOXA2	homeobox A2
Stomach Neoplasms	APC	adenomatous polyposis coli
Stomach Tumor	ACHE	acetylcholinesterase
Gastric Cancer	AR	androgen Receptor
Gastric Carcinoma	CYP2A6	aytochrome P450 2A6
Gastric Cancer	ESR1	estrogen Receptor
Gastric Cancer	GLO1	glyoxalase I
Gastric Carcinoma	MMP9	matrix metalloproteinase 9
Gastric Cancer	NR3C1	glucocorticoid receptor
Gastric Cancer	PLA2G2A	phospholipase A2 group IIA
Stomach Tumor	PLK2	polo-Like Kinase 2
Gastrointestinal Neoplasms	APC	adenomatous polyposis coli
Stomach Neoplasms	HRAS	harvey rat sarcoma viral oncogene homolog
Stomach Neoplasms	APEX1	APEX nuclease (multifunctional DNA repair enzyme) 1
Stomach Neoplasms	BIRC2	baculoviral IAP repeat containing 2
Stomach Neoplasms	HSPA8	heat shock 70kDa protein 8
Stomach Neoplasms	HSPB1	heat shock 27kDa protein 1
Stomach Neoplasms	BIRC5	baculoviral IAP repeat containing 5
Stomach Neoplasms	HSPD1	heat shock 60kDa protein 1 (chaperonin)
Stomach Neoplasms	APOA1	apolipoprotein A-I
Stomach Neoplasms	HTR1A	5-hydroxytryptamine (serotonin) receptor 1A, G protein-coupled
Stomach Ulcer	IAPP	islet amyloid polypeptide

Stomach Neoplasms	ICAM2	intercellular adhesion molecule 2
Stomach Neoplasms	ID4	inhibitor of DNA binding 4, dominant negative helix-loop-helix protein
Stomach Neoplasms	IDH3B	isocitrate dehydrogenase 3 (NAD+) beta
Stomach Neoplasms	IGFBP3	insulin-like growth factor binding protein 3
Stomach Neoplasms	IGFBP7	insulin-like growth factor binding protein 7
Stomach Ulcer	IL1B	interleukin 1, beta
Stomach Neoplasms	IL1B	interleukin 1, beta
Gastritis, Atrophic	IL1B	interleukin 1, beta
Stomach Neoplasms	IL1RN	interleukin 1 receptor antagonist
Stomach Neoplasms	IL6	interleukin 6
Stomach Neoplasms	IL6R	interleukin 6 receptor
Gastroesophageal Reflux	CXCL8	chemokine (C-X-C motif) ligand 8
Stomach Neoplasms	CXCL8	chemokine (C-X-C motif) ligand 8
Stomach Neoplasms	TNFRSF9	tumor necrosis factor receptor superfamily, member 9
Stomach Neoplasms	ING1	inhibitor of growth family, member 1
Stomach Neoplasms	IRF1	interferon regulatory factor 1
Stomach Neoplasms	ITGA5	integrin, alpha 5 (fibronectin receptor, alpha polypeptide)
Stomach Neoplasms	JUN	jun proto-oncogene
Stomach Neoplasms	AREG	amphiregulin
Stomach Neoplasms	KISS1	KiSS-1 metastasis-suppressor
Gastrointestinal Stromal Tumors	KIT	v-kit Hardy-Zuckerman 4 feline sarcoma viral oncogene homolog
Stomach Neoplasms	KRAS	Kirsten rat sarcoma viral oncogene homolog
Stomach Neoplasms	KRT8	keratin 8, type II
Stomach Neoplasms	RHOA	ras homolog family member A
Stomach Ulcer	LEP	leptin
Stomach Neoplasms	LGALS3	lectin, galactoside-binding, soluble, 3
Stomach Neoplasms	FADS1	fatty acid desaturase 1
Gastrointestinal Hemorrhage	LTF	lactotransferrin
Stomach Neoplasms	M6PR	mannose-6-phosphate receptor (cation dependent)
Stomach Neoplasms	SMAD4	SMAD family member 4
Stomach Neoplasms	MARK1	MAP/microtubule affinity-regulating kinase 1
Gastrinoma	MEN1	multiple endocrine neoplasia I
Stomach Neoplasms	MET	MET proto-oncogene, receptor tyrosine kinase
Stomach Neoplasms	MLF1	myeloid leukemia factor 1
Stomach Neoplasms	KMT2A	lysine (K)-specific methyltransferase 2A
Stomach Ulcer	MMP9	matrix metallopeptidase 9
Stomach Neoplasms	MMP10	matrix metallopeptidase 10
Stomach Neoplasms	MSX1	msh homeobox 1
Stomach Neoplasms	MT2A	metallothionein 2A
Stomach Neoplasms	MTHFR	methylenetetrahydrofolate reductase (NAD(P)H)
Gastrointestinal Diseases	MTR	5-methyltetrahydrofolate-homocysteine methyltransferase
Stomach Neoplasms	MUC6	mucin 6, oligomeric mucus/gel-forming

Stomach Neoplasms	MUTYH	mutY homolog
Stomach Neoplasms	MX1	MX dynamin-like GTPase 1
Stomach Neoplasms	MYC	v-myc avian myelocytomatosis viral oncogene homolog
Gastrointestinal Diseases	MYLK	myosin light chain kinase
Stomach Neoplasms	NDUFA2	NADH dehydrogenase (ubiquinone) 1 alpha subcomplex, 2, 8kDa
Stomach Neoplasms	NDUFS1	NADH dehydrogenase (ubiquinone) Fe-S protein 1, 75kDa (NADH-coenzyme Q reductase)
Stomach Neoplasms	NDUFV1	NADH dehydrogenase (ubiquinone) flavoprotein 1, 51kDa
Gastrointestinal Diseases	NFE2L2	nuclear factor, erythroid 2-like 2
Stomach Ulcer	NOS2	nitric oxide synthase 2, inducible
Stomach Ulcer	NOS3	nitric oxide synthase 3 (endothelial cell)
Stomach Neoplasms	NOS3	nitric oxide synthase 3 (endothelial cell)
Stomach Neoplasms	NPM1	nucleophosmin (nucleolar phosphoprotein B23, numatrin)
Stomach Neoplasms	NT5E	5'-nucleotidase, ecto (CD73)
Stomach Neoplasms	ALDH7A1	aldehyde dehydrogenase 7 family, member A1
Stomach Neoplasms	PA2G4	proliferation-associated 2G4, 38kDa
Stomach Neoplasms	SERPINE1	serpin peptidase inhibitor, clade E (nexin, plasminogen activator inhibitor type 1), member 1
Stomach Neoplasms	SERPINB2	serpin peptidase inhibitor, clade B (ovalbumin), member 2
Stomach Neoplasms	PAX6	paired box 6
Stomach Neoplasms	COPS7A	COP9 signalosome subunit 7A
Stomach Neoplasms	UBXN1	UBX domain protein 1
Stomach Neoplasms	ZNF593	zinc finger protein 593
Stomach Neoplasms	PLCE1	phospholipase C, epsilon 1
Stomach Neoplasms	GMPR2	guanosine monophosphate reductase 2
Stomach Neoplasms	C11ORF73	chromosome 11 open reading frame 73
Gastrointestinal Stromal Tumors	PDGFRA	platelet-derived growth factor receptor, alpha polypeptide
Stomach Neoplasms	NBAS	neuroblastoma amplified sequence
Stomach Neoplasms	PDHA1	pyruvate dehydrogenase (lipoamide) alpha 1
Stomach Neoplasms	POLR3K	polymerase (RNA) III (DNA directed) polypeptide K, 12.3 kDa
Stomach Ulcer	GHRL	ghrelin/obestatin prepropeptide
Stomach Neoplasms	WWOX	WW domain containing oxidoreductase
Stomach Neoplasms	PGAM1	phosphoglycerate mutase 1 (brain)
Stomach Neoplasms	PHB	prohibitin
Stomach Neoplasms	SERPINA1	serpin peptidase inhibitor, clade A (alpha-1 antiproteinase, antitrypsin), member 1
Stomach Neoplasms	PIK3CA	phosphatidylinositol-4,5-bisphosphate 3-kinase, catalytic subunit alpha
Stomach Neoplasms	PLAGL1	pleiomorphic adenoma gene-like 1
Stomach Neoplasms	PLAU	plasminogen activator, urokinase
Stomach Neoplasms	PTOV1	prostate tumor overexpressed 1
Gastrointestinal Diseases	POMC	proopiomelanocortin
Stomach Neoplasms	KRT20	keratin 20, type I
Stomach Neoplasms	PPARG	peroxisome proliferator-activated receptor gamma

Stomach Neoplasms	XAF1	XIAP associated factor 1
Stomach Neoplasms	PPIA	peptidylprolyl isomerase A (cyclophilin A)
Stomach Neoplasms	PPIC	peptidylprolyl isomerase C (cyclophilin C)
Stomach Neoplasms	RNF43	ring finger protein 43
Gastrointestinal Hemorrhage	AVP	arginine vasopressin
Stomach Neoplasms	PPP2R1A	protein phosphatase 2, regulatory subunit A, alpha
Stomach Neoplasms	PPP2R4	protein phosphatase 2A activator, regulatory subunit 4
Stomach Neoplasms	PRR5-ARHGAP8	PRR5-ARHGAP8 readthrough
Stomach Neoplasms	PREP	prolyl endopeptidase
Stomach Neoplasms	PRKAB1	protein kinase, AMP-activated, beta 1 non-catalytic subunit
Stomach Neoplasms	CARKD	carbohydrate kinase domain containing
Stomach Neoplasms	CHFR	checkpoint with forkhead and ring finger domains, E3 ubiquitin protein ligase
Stomach Neoplasms	PRKCB	protein kinase C, beta
Stomach Neoplasms	MAPK1	mitogen-activated protein kinase 1
Stomach Neoplasms	MAPK3	mitogen-activated protein kinase 3
Stomach Neoplasms	MAPK8	mitogen-activated protein kinase 8
Stomach Neoplasms	PRNP	prion protein
Stomach Neoplasms	GKN1	gastrokine 1
Stomach Neoplasms	KLK10	kallikrein-related peptidase 10
Stomach Neoplasms	EXOSC5	exosome component 5
Stomach Ulcer	PTGS1	prostaglandin-endoperoxide synthase 1 (prostaglandin G/H synthase and cyclooxygenase)
Stomach Ulcer	PTGS2	prostaglandin-endoperoxide synthase 2 (prostaglandin G/H synthase and cyclooxygenase)
Stomach Neoplasms	PTGS2	prostaglandin-endoperoxide synthase 2 (prostaglandin G/H synthase and cyclooxygenase)
Gastroesophageal Reflux	PTGS2	prostaglandin-endoperoxide synthase 2 (prostaglandin G/H synthase and cyclooxygenase)
Stomach Neoplasms	RANBP10	RAN binding protein 10
Gastrointestinal Diseases	CHD8	chromodomain helicase DNA binding protein 8
Stomach Neoplasms	PTPRF	protein tyrosine phosphatase, receptor type, F
Stomach Neoplasms	PTPRG	protein tyrosine phosphatase, receptor type, G
Stomach Neoplasms	KMT2C	lysine (K)-specific methyltransferase 2C
Stomach Neoplasms	RAD23A	RAD23 homolog A (S. cerevisiae)
Stomach Neoplasms	RARB	retinoic acid receptor, beta
Stomach Neoplasms	RARRES1	retinoic acid receptor responder (tazarotene induced) 1
Gastroesophageal Reflux	GER	Gastroesophageal reflux
Stomach Neoplasms	HRH4	histamine receptor H4
Stomach Neoplasms	RBP1	retinol binding protein 1, cellular
Stomach Neoplasms	CCND1	cyclin D1
Stomach Neoplasms	RBP4	retinol binding protein 4, plasma
Stomach Neoplasms	RGS2	regulator of G-protein signaling 2

Stomach Neoplasms	RORA	RAR-related orphan receptor A
Stomach Neoplasms	RPL13	ribosomal protein L13
Stomach Neoplasms	RPL15	ribosomal protein L15
Stomach Neoplasms	RPL18	ribosomal protein L18
Stomach Neoplasms	RPS6	ribosomal protein S6
Stomach Neoplasms	RPS15	ribosomal protein S15
Stomach Neoplasms	RPS19	ribosomal protein S19
Stomach Neoplasms	RPS21	ribosomal protein S21
Stomach Neoplasms	RPS26	ribosomal protein S26
Stomach Neoplasms	RXRB	retinoid X receptor, beta
Stomach Neoplasms	BDNF	brain-derived neurotrophic factor
Stomach Neoplasms	BID	BH3 interacting domain death agonist
Stomach Neoplasms	BLVRB	biliverdin reductase B
Stomach Neoplasms	MRPS11	mitochondrial ribosomal protein S11
Stomach Neoplasms	BMP2	bone morphogenetic protein 2
Stomach Neoplasms	SLC1A2	solute carrier family 1 (glial high affinity glutamate transporter), member 2
Stomach Neoplasms	BMP7	bone morphogenetic protein 7
Stomach Neoplasms	SNAI1	snail family zinc finger 1
Stomach Neoplasms	SNRPB	small nuclear ribonucleoprotein polypeptides B and B1
Stomach Neoplasms	BNIP3	BCL2/adenovirus E1B 19kDa interacting protein 3
Stomach Neoplasms	SOD2	superoxide dismutase 2, mitochondrial
Stomach Neoplasms	SPRR2A	small proline-rich protein 2A
Stomach Neoplasms	SREBF2	sterol regulatory element binding transcription factor 2
Esophageal and Gastric Varices	SST	somatostatin
Stomach Neoplasms	STAT3	signal transducer and activator of transcription 3 (acute-phase response factor)
Gastroesophageal Reflux	TAC1	tachykinin, precursor 1
Stomach Neoplasms	TBX3	T-box 3
Stomach Neoplasms	ACTC1	actin, alpha, cardiac muscle 1
Stomach Neoplasms	TFAP2C	transcription factor AP-2 gamma (activating enhancer binding protein 2 gamma)
Stomach Neoplasms	TGFA	transforming growth factor, alpha
Stomach Neoplasms	THBD	thrombomodulin
Stomach Neoplasms	TIMP3	TIMP metallopeptidase inhibitor 3
Stomach Ulcer	TNF	tumor necrosis factor
Stomach Neoplasms	TNF	tumor necrosis factor
Stomach Neoplasms	TP53	tumor protein p53
Stomach Neoplasms	TPM3	tropomyosin 3
Stomach Neoplasms	TWIST1	twist family bHLH transcription factor 1
Stomach Neoplasms	TYMS	thymidylate synthetase
Stomach Neoplasms	UMPS	uridine monophosphate synthetase
Stomach Ulcer	VEGFA	vascular endothelial growth factor A
Stomach Ulcer	VGF	VGF nerve growth factor inducible

Stomach Neoplasms	XRCC1	X-ray repair complementing defective repair in Chinese hamster cells 1
Stomach Neoplasms	XRCC3	X-ray repair complementing defective repair in Chinese hamster cells 3
Stomach Neoplasms	CA1	carbonic anhydrase I
Stomach Neoplasms	CA2	carbonic anhydrase II
Stomach Neoplasms	ZNF177	zinc finger protein 177
Stomach Neoplasms	FSD1	fibronectin type III and SPRY domain containing 1
Stomach Neoplasms	FAT4	FAT atypical cadherin 4
Stomach Neoplasms	EPHX3	epoxide hydrolase 3
Stomach Neoplasms	PSCA	prostate stem cell antigen
Gastric Antral Vascular Ectasia	CTC1	CTS telomere maintenance complex component 1
Stomach Neoplasms	PUS1	pseudouridylate synthase 1
Stomach Neoplasms	ULBP2	UL16 binding protein 2
Stomach Neoplasms	TAF15	TAF15 RNA polymerase II, TATA box binding protein (TBP)-associated factor, 68kDa
Stomach Neoplasms	URM1	ubiquitin related modifier 1
Stomach Neoplasms	SYMPK	symplekin
Stomach Neoplasms	MIA	melanoma inhibitory activity
Stomach Neoplasms	ARID1A	AT rich interactive domain 1A (SWI-like)
Stomach Neoplasms	REG4	regenerating islet-derived family, member 4
Stomach Neoplasms	CASP8	caspase 8, apoptosis-related cysteine peptidase
Stomach Neoplasms	CASP10	caspase 10, apoptosis-related cysteine peptidase
Stomach Neoplasms	ARFGAP2	ADP-ribosylation factor GTPase activating protein 2
Stomach Neoplasms	ZNF559	zinc finger protein 559
Stomach Neoplasms	KISS1R	KISS1 receptor
Stomach Neoplasms	SPZ1	spermatogenic leucine zipper 1
Stomach Ulcer	CAT	catalase
Stomach Neoplasms	TUBA1C	tubulin, alpha 1c
Stomach Neoplasms	ITGA8	integrin, alpha 8
Stomach Neoplasms	CST7	cystatin F (leukocystatin)
Stomach Neoplasms	CAV1	caveolin 1, caveolae protein, 22kDa
Stomach Neoplasms	PPAP2A	phosphatidic acid phosphatase type 2A
Stomach Neoplasms	RUNX3	runt-related transcription factor 3
Stomach Neoplasms	AKR1C3	aldo-keto reductase family 1, member C3
Stomach Neoplasms	IRS2	insulin receptor substrate 2
Stomach Neoplasms	TNFSF9	tumor necrosis factor (ligand) superfamily, member 9
Stomach Neoplasms	SUCLG1	succinate-CoA ligase, alpha subunit
Stomach Ulcer	CCK	cholecystokinin
Gastrointestinal Diseases	CCK	cholecystokinin
Stomach Neoplasms	FCGBP	Fc fragment of IgG binding protein
Stomach Neoplasms	SELENBP1	selenium binding protein 1
Stomach Neoplasms	ZNF160	zinc finger protein 160
Stomach Neoplasms	RRP9	ribosomal RNA processing 9, small subunit (SSU) processome component, homolog (yeast)

Stomach Neoplasms	AURKB	aurora kinase B
Stomach Neoplasms	IL32	interleukin 32
Stomach Neoplasms	WDR46	WD repeat domain 46
Gastrointestinal Neoplasms	GDF15	growth differentiation factor 15
Stomach Neoplasms	CD44	CD44 molecule (Indian blood group)
Stomach Neoplasms	TMEM63A	transmembrane protein 63A
Stomach Neoplasms	MTSS1	metastasis suppressor 1
Stomach Neoplasms	SCRN1	secernin 1
Gastrointestinal Diseases	KEAP1	kelch-like ECH-associated protein 1
Stomach Neoplasms	CDH1	cadherin 1, type 1, E-cadherin (epithelial)
Stomach Neoplasms	CDH2	cadherin 2, type 1, N-cadherin (neuronal)

Supplementary S3 Herbs belonged to heart meridian

Herb Name	Pinyin	Meridian	Property and Flavor
Acanthopanacis Senticosi Radix Et Rhizoma Seu Caulis	Ciwujia	spleen, kidney, heart	pungent, little bitter, temperature
Aconiti Radix	Chuanwu	heart, liver, kidney, spleen	pungent, bitter, hot, large toxic
Aconiti Kusnezoffii Radix	Caowu	heart, liver, kidney, spleen	pungent, bitter, hot, large toxic
Aconiti Kusnezoffii Radix Cocta	Zhicaowu	heart, liver, kidney, spleen	pungent, bitter, hot, toxic
Aconiti Lateralis Radix Praeparae4	Fuzi	heart, kidney, spleen	pungent, sweet, large hot, toxic
Aconiti Radix Cocta	Zhichaunwu	heart, liver, kidney, spleen	pungent, bitter, hot, toxic
Acori Tatarinowii Rhizoma	Shichangpu	heart, stomach	pungent, bitter, temperature
Agrimoniae Herba	Xianhecao	heart, liver	bitter, astringent, natured
Akebiae Caulis	Mutong	heart, small intestine, bladder	bitter, cold
Albiziae Cortex	Hehuanpi	heart, liver, lung	sweet, natured
Albiziae Flos	Hehuanhua	heart, liver	sweet, natured
Allii Macrostemonis Bulbus	Xiebai	heart, lung, stomach, large intestine	pungent, bitter, temperature
Ampelopsis Radix	Bailian	heart, stomach	bitter, little cold
Andrographis Herba	Chaunxinlian	heart, lung, large intestine, bladder	bitter, cold
Angelicae Sinensis Radix	Danggui	liver, heart, spleen	sweet, pungent, temperature
Arnebiae Radix	Zicao	heart, liver	sweet, salty, cold
Asari Radix Et Rhizoma	Xixin	heart, lung, kidney	pungent, temperature
Bambusae Caulis In Taenias	Zhuru	lung, stomach, heart, gallbladder	sweet, little cold
Bambusae Concretio Silicea	Tianzhuhuang	heart, liver	sweet, cold
Baphicacanthis Cusiae Rhizoma Et Radix	Nanbanlangen	heart, stomach	bitter, cold
Benzoinum	Anxixiang	heart, spleen	pungent, bitter, natured
Borneolum Syntheticum	Bingpian	heart, spleen, lung	pungent, bitter, little cold
Campsis Flos	Lingxiaohua	liver, pericardium	sweet, sour, cold
Capsici Fructus	Lajiao	heart, spleen	pungent, hot
Carthami Flos	Honghua	heart, liver	pungent, temperature
Catechu	Ercha	lung, heart	bitter, astringent, little cold
Choerospondiatis Fructus	Guangzao	heart, liver	sweet, sour, natured
Chrysanthemi Indici Flos	Yejuhua	liver, heart	bitter, pungent, little cold
Chuanxiong Rhizoma	Chuanxiong	liver, gallbladder, pericardium	pungent, temperature
Cinnamomi Cortex	Rougui	kidney, spleen, heart, liver	pungent, sweet, large hot
Cinnamomi Ramulus	Guizhi	heart, lung, bladder	pungent, sweet, temperature
Cirsii Japonici Herba	Daji	heart, liver	sweet, bitter, cool
Cirsii Japonici Herba	Dajitan	heart, liver	bitter, astringent, cool

Carbonisata			
Clematidis Armandii Caulis	Chuanmutong	heart, small intestine, bladder	bitter, cold
Coptidis Rhizoma	Huanglian	heart, spleen, stomach, liver, gallbladder, large intestine	bitter, cold
Coriolus	Yunzhi	heart, spleen, liver, kidney	sweet, natured
Corydalis Bungeanae Herba	Kudiding	heart, liver, large intestine	bitter, cold
Croci Stigma	Xihonghua	heart, liver	sweet, natured
Curcumae Radix	Yujin	liver, heart, lung	pungent, bitter, cold
Dianthi Herba	Qumai	heart, small intestine	bitter, cold
Dichroae Radix	Changshan	lung, liver, heart	bitter, pungent, cold, toxic
Draconis Sanguis	Xuejie	heart, liver	sweet, salty, natured
Ephedrae Radix E T Rhizoma	Mahuanggen	heart, lung	sweet, astringent, natured
Erigerontis Herba	Dengzhanxixin (Dengzhanhua)	heart, liver	pungent, little bitter, temperature
Fibraureae Caulis	Huangteng	heart, liver	bitter, cold
Forsythiae Fructus	Lianqiao	lung, heart, small intestine	bitter, little cold
Fritillariae Cirrhosae Bulbus	Chuanbeimu	lung, heart	bitter, sweet, little cold
Fritillariae Hupehensis Bulbus	Hubeibeimu	lung, heart	little bitter, cool
Fritillariae Pallidiflorae Bulbus	Yibeimu	lung, heart	bitter, sweet, little cold
Fritillariae Thunbergii Bulbus	Zhebeimu	lung, heart	bitter, cold
Fritillariae Ussuriensis Bulbus	Pingbeimu	lung, heart	bitter, sweet, little cold
Ganoderma	Lingzhi	heart, lung, liver, kidney	sweet, natured
Gardeniae Fructus	Zhizi	heart, lung, Sanjiao channel	bitter, cold
Gardeniae Fructus Praeparatus	Jiaozhizi	heart, lung, Sanjiao channel	bitter, cold
Ginkgo Folium	Yinxingye	heart, lung	sweet, bitter, astringent, natured
Ginseng Radix Et Rhizoma	Renshen	spleen, lung, heart, kidney	sweet, little bitter, little temperature
Ginseng Radix Et Rhizoma Rubra	Hongshen	spleen, lung, heart, kidney	sweet, little bitter, temperature
Glycyrrhizae Radix Et Rhizoma	Gancao	heart, lung, spleen, stomach	sweet, natured
Glycyrrhizae Radix Et Rhizoma Praeparata Cum Melle	Zhigancao	heart, lung, spleen, stomach	sweet, natured
Hippophae Fructus	Shaji	spleen, stomach, lung, heart	sour, astringent, temperature
Hyoscyami Semen	Tianxianzi	heart, stomach, liver	bitter, pungent, temperature, large toxic
Isatidis Folium	Daqingye	heart, stomach	bitter, cold
Isatidis Radix	Banlangen	heart, stomach	bitter, cold
Jujubae Fructus	Dazao	heart, spleen, stomach	sweet, temperature
Junci Medulla	Dengxincao	heart, lung, small intestine	sweet, bland, little cold
Leonuri Fructus	Chongweizi	pericardium, liver	pungent, bitter, little cold
Leonuri Herba	Yimucao	liver, pericardium, bladder	bitter, pungent, little cold
Lilii Bulbus	Baihe	heart, lung	sweet, cold
Liriopes Radix	Shanmaidong	heart, lung, stomach	sweet, little bitter, little cold
Lobeliae Chinensis Herba	Banbianlian	heart, small intestine, lung	pungent, natured
Longan Arillus	Longyanrou	heart, spleen	sweet, temperature

Lonicerae Flos	Shanyinhua	lung, heart, stomach	sweet, cold
Lonicerae Japonicae Flos	Jinyinhua	lung, heart, stomach	sweet, cold
Lophatheri Herba	Danzhuye	heart, stomach, small intestine	sweet, bland, cold
Mori Fructus	Sangshen	heart, liver, kidney	sweet, sour, cold
Moschus	Shexiang	heart, spleen	pungent, temperature
Moutan Cortex	Mudanpi	heart, liver, kidney	bitter, pungent, little cold
Myrrha	Moyao	heart, liver, spleen	pungent, bitter, natured
Nelumbinis Plumula	Lianzixin	heart, kidney	bitter, cold
Nelumbinis Semen	Lianzi	spleen, kidney, heart	sweet, astringent, natured
Nelumbinis Stamen	Lianxu	heart, kidney	sweet, astringent, natured
Olibanum	Ruxiang	heart, liver, spleen	pungent, bitter, temperature
Ophiopogonis Radix	Maidong	heart, lung, stomach	sweet, little bitter, little cold
Panacis Quinquefolii Radix	Xiyangshen	heart, lung, kidney	sweet, little bitter, cool
Periplocae Cortex	Xiangjiapi	liver, kidney, heart	pungent, bitter, temperature, toxic
Persicae Ramulus	Taozhi	heart, liver	bitter, natured
Persicae Semen	Taoren	heart, liver, large intestine	bitter, sweet, natured
Physochlainae Radix	Huashanshen	lung, heart	sweet, little bitter, temperature, toxic
Platycladi Semen	Boziren	heart, kidney, large intestine	sweet, natured
Polygalae Radix	Yuanzhi	heart, kidney, lung	bitter, pungent, temperature
Polygoni Multiflori Caulis	Shouwuteng	heart, liver	sweet, natured
Polygoni Multiflori Radix	Heshouwu	liver, heart, kidney	bitter, sweet, astringent, little temperature
Polygoni Multiflori Radix Praeparata	Zhiheshouwu	liver, heart, kidney	bitter, sweet, astringent, little temperature
Polygoni Tinctorii Folium	Liaodaqingye	heart, stomach	bitter, cold
Poria	Fuling	heart, lung, spleen, kidney	sweet, bland, natured
Rehmanniae Radix	Dihuang	heart, liver, kidney	sweet, bitter, cold
Rhei Radix Et Rhizoma	Dahuang	spleen, stomach, large intestine, liver, pericardium	bitter, cold
Rhodiolae Crenulatae Radix Et Rhizoma	Hongjingtian	lung, heart	sweet, bitter, natured
Salviae Miltiorrhizae Radix Et Rhizoma	Danshen	heart, liver	bitter, little cold
Santali Albi Lignum	Tanxiang	spleen, stomach, heart, lung	pungent, temperature
Sappan Lignum	Sumu	heart, liver, spleen	sweet, salty, natured
Sarcandrae Herba	Zhongjiefeng	heart, liver	bitter, pungent, natured
Schisandrae Chinensis Fructus	Wuweizi	lung, heart, kidney	sour, sweet, temperature
Schisandrae Sphenantherae Fructus	Nanwuweizi	lung, heart, kidney	sour, sweet, temperature
Selaginellae Herba	Juanbai	liver, heart	pungent, natured
Sophorae Flavescentis Radix	Kushen	heart, liver, stomach, large intestine, bladder	bitter, cold

Styrax	Suhexiang	heart, spleen	pungent, temperature
Tamaricis Cacumen	Xiheliu	heart, lung, stomach	sweet, pungent, natured
Trachelospermi Caulis E T Folium	Luoshiteng	heart, liver, kidney	bitter, little cold
Typhae Pollen	Puhuang	liver, pericardium	sweet, natured
Uncariae Ramulus C U M Uncis	Gouteng	liver, pericardium	sweet, cool
Vignae Semen	Chixiaodou	heart, small intestine	sweet, sour, natured
Violae Herba	Zihuadiding	heart, liver	bitter, pungent, cold
Zingiberis Rhizoma	Ganjiang	spleen, stomach, kidney, heart, lung	pungent, hot
Ziziphi Spinosae Semen	Suanzaoren	liver, gallbladder, heart	sweet, sour, natured

Supplementary S4 Herbs belonged to stomach meridian

Herb Name	Pinyin	Meridian	Property and Flavor
Abri Herba	Jigucao	liver, stomach	sweet, little bitter, cool
Acori Tatarinowii Rhizoma	Shichangpu	heart, stomach	pungent, bitter, temperature
Adenophorae Radix	Nanshashen	lung, stomach	sweet, little cold
Aesculi Semen	Suoluozi	liver, stomach	sweet, temperature
Ailanthi Cortex	Chunpi	large intestine, stomach, liver	bitter, astringent, cold
Akebiae Fructus	Yuzhizi	liver, gallbladder, stomach, bladder	bitter, cold
Allii Sativi Bulbus	Dasuan	spleen, stomach, lung	pungent, temperature
Allii Macrostemonis Bulbus	Xiebai	heart, lung, stomach, large intestine	pungent, bitter, temperature
Aloe	Luhui	liver, stomach, large intestine	bitter, cold
Alpiniae Katsumadai Semen	Caodoukou	spleen, stomach	pungent, temperature
Alpiniae Officinarum Rhizoma	Gaoliangjiang	spleen, stomach	pungent, hot
Amomi Fructus	Sharen	spleen, stomach, kidney	pungent, temperature
Amomi Fructus Rotundus	Doukou	lung, spleen, stomach	pungent, temperature
Ampelopsis Radix	Bailian	heart, stomach	bitter, little cold
Ampelopsis Radix	Baiwei	stomach, liver, kidney	bitter, salty, cold
Anemarrhenae Rhizoma	Zhimu	lung, stomach, kidney	bitter, sweet, cold
Angelicae Dahuricae Radix	Baizhi	stomach, large intestine, lung	pungent, temperature
Anisi Stellati Fructus	Bajiaohuixiang	liver, kidney, spleen, stomach	pungent, temperature
Aquilariae Lignum Resinatum	Chenxiang	spleen, stomach, kidney	pungent, bitter, little temperature
Arctii Fructus	Niubangzi	lung, stomach	pungent, bitter, cold
Arecae Pericarpium	Dafupi	spleen, stomach, large intestine, small intestine	pungent, little temperature
Arecae Semen	Binglang	stomach, large intestine	bitter, pungent, temperature
Arecae Semen Tostum	Jiaobinglang	stomach, large intestine	bitter, pungent, temperature
Artemisiae Scopariae Herba	Yinchen	spleen, stomach, liver, gallbladder	bitter, pungent, little cold
Atractylodis Macrocephalae Rhizoma	Baizhu	spleen, stomach	bitter, sweet, temperature
Atractylodis Rhizoma	Cangzhu	spleen, stomach, liver	pungent, bitter, temperature
Aucklandiae Radix	Muxiang	spleen, stomach, large intestine, Sanjiao channel, gallbladder	pungent, bitter, temperature
Aurantii Fructus	Zhike	spleen, stomach	bitter, pungent, sour, little cold
Aurantii Fructus Immaturus	Zhishi	spleen, stomach	bitter, pungent, sour, little cold
Bambusae Caulis In Taenias	Zhuru	lung, stomach, heart, gallbladder	sweet, little cold
Baphicacanthis Cusiae Rhizoma Et Radix	Nanbanlangen	heart, stomach	bitter, cold
Berberidis Radix	Sankezhen	liver, stomach, large intestine	bitter, cold, toxic
Bletillae Rhizoma	Baiji	lung, liver, stomach	bitter, sweet, astringent, little cold
Callicarpae Formosanae Folium	Zizhuye	liver, lung, stomach	bitter, astringent, cool
Canarii Fructus	Qingguo	lung, stomach	sweet, sour, natured
Canavaliae Semen	Daodou	stomach, kidney	sweet, temperature

Cannabis Semen	Huomaren	spleen, stomach, large intestine	sweet, natured
Carotae Fructus	Nanheshi	spleen, stomach	bitter, pungent, natured, little toxic
Carpesh Fructus	Heshi	spleen, stomach	bitter, pungent, natured, little toxic
Caryophylli Flos	Dingxiang	spleen, stomach, lung, kidney	pungent, temperature
Caryophyllifructus	Mudingxiang	spleen, stomach, lung, kidney	pungent, temperature
Chelidonii Herba	Baiqucai	lung, stomach	bitter, cool, toxic
Cichorii Herba Cichorii Radix	Juju	liver, gallbladder, stomach	little bitter, salty, cool
Cimicifugae Rhizoma	Shengma	lung, spleen, stomach, large intestine	pungent, little sweet, little cold
Citri Reticulatae Pericarpium Viride	Qingpi	liver, gallbladder, stomach	bitter, pungent, temperature
Citri Sarcodactylis Fructus	Foshou	liver, spleen, stomach, lung	pungent, bitter, sour, temperature
Coicis Semen	Yiyiren	spleen, stomach, lung	sweet, bland, cool
Commelinae Herba	Yazhicao	lung, stomach, small intestine	sweet, bland, cold
Coptidis Rhizoma	Huanglian	heart, spleen, stomach, liver, gallbladder, large intestine	bitter, cold
Crataegifructus	Shanzha	spleen, stomach, liver	sour, sweet, little temperature
Crotonis Semen Pulveratum	Badoushuang	stomach, large intestine	pungent, hot, large toxic
Crotonis Fructus	Badou	stomach, large intestine	pungent, hot, large toxic
Cynanchi Paniculati Radix Et Rhizoma	Xuchangqing	liver, stomach	pungent, temperature
Dendrobii Caulis	Shihu	stomach, kidney	sweet, little cold
Dictamni Cortex	Baixianpi	spleen, stomach, bladder	bitter, cold
Dryopteridis Crassirhizomatis Rhizoma	Mianmaguanzhong	liver, stomach	bitter, little cold, little toxic
Echinopsis Radix	Yuzhouloulu	stomach	bitter, cold
Eriobotryae Folium	Pipaye	lung, stomach	bitter, little cold
Erycibes Caulis	Dinggongteng	liver, spleen, stomach	pungent, temperature, little toxic
Euodiae Fructus	Wuzhuyu	liver, spleen, stomach, kidney	pungent, bitter, hot, little toxic
Eupatorii Herba	Peilan	spleen, stomach, lung	pungent, natured
Ferulae Resina	Awei	spleen, stomach	bitter, pungent, temperature
Foeniculi Fructus	Xiaohuixiang	liver, kidney, spleen, stomach	pungent, temperature
Gentianae Macrophyllae Radix	Qinjiao	stomach, liver, gallbladder	pungent, bitter, natured
Ginseng Folium	Renshenye	lung, stomach	bitter, sweet, cold
Gleditsiae Spina	Zaojiaoci	liver, stomach	pungent, temperature
Glehniae Radix	Beishashen	lung, stomach	sweet, little bitter, little cold
Glycyrrhizae Radix Et Rhizoma	Gancao	heart, lung, spleen, stomach	sweet, natured
Glycyrrhizae Radix Et Rhizoma Praeparata Cum Melle	Zhigancao	heart, lung, spleen, stomach	sweet, natured
Hippophae Fructus	Shaji	spleen, stomach, lung, heart	sour, astringent, temperature
Hordei Fructus Germinatus	Maiya	spleen, stomach	sweet, natured
Hyoscyami Semen	Tianxianzi	heart, stomach, liver	bitter, pungent, temperature, large toxic
Ilicis Rotundae Cortex	Jiubiying	lung, stomach, large intestine, liver	bitter, cold
Imperatae Rhizoma	Baimaogen	lung, stomach, bladder	sweet, cold

Inulae Flos	Xuanfuhua	lung, spleen, stomach, large intestine	bitter, pungent, salty, little temperature
Isatidis Folium	Daqingye	heart, stomach	bitter, cold
Isatidis Radix	Banlangen	heart, stomach	bitter, cold
Jujubae Fructus	Dazao	heart, spleen, stomach	sweet, temperature
Kaempferiae Rhizoma	Shannai	stomach	pungent, temperature
Kaki Calyx	Shidi	stomach	bitter, astringent, natured
Lablab Semen Album	Baibiandou	spleen, stomach	sweet, little temperature
Liriopes Radix	Shanmaidong	heart, lung, stomach	sweet, little bitter, little cold
Litseae Fructus	Bichengqie	spleen, stomach, kidney, bladder	pungent, temperature
Lonicerae Flos	Shanyinhua	lung, heart, stomach	sweet, cold
Lonicerae Japonicae Caulis	Rendongteng	lung, stomach	sweet, cold
Lonicerae Japonicae Flos	Jinyinhua	lung, heart, stomach	sweet, cold
Lophatheri Herba	Danzhuye	heart, stomach, small intestine	sweet, bland, cold
Luffae Fructus Retinervus	Sigualuo	lung, stomach, liver	sweet, natured
Magnoliae Flos	Xinyi	lung, stomach	pungent, temperature
Magnoliae Officinalis Cortex	Houpu	spleen, stomach, lung, large intestine	bitter, pungent, temperature
Magnoliae Officinalis Flos	Houpuhua	spleen, stomach	bitter, little temperature
Mahoniae Caulis	Gonglaomu	liver, stomach, large intestine	bitter, cold
Meliae Cortex	Kulianpi	liver, spleen, stomach	bitter, cold, toxic
Menispermi Rhizoma	Beidougen	lung, stomach, large intestine	bitter, cold, little toxic
Microctis Folium	Buzhaye	spleen, stomach	little sour, cool
Mirabilitum Praeparatum	Xiguashuang	lung, stomach, large intestine	salty, cold
Momordicae Semen	Mubiezi	liver, spleen, stomach	bitter, little sweet, cool, toxic
Moslae Herba	Xiangru	lung, stomach	pungent, little temperature
Mume Flos	Meihua	liver, stomach, lung	little sour, natured
Murrayae Folium Et Cacumen	Jiulixiang	liver, stomach	pungent, little bitter, temperature, little toxic
Myristicae Semen	Roudoukou	spleen, stomach, large intestine	pungent, temperature
Nardostachyos Radix Et Rhizoma	Gansong	spleen, stomach	pungent, sweet, temperature
Nelumbinis Folium	Heye	liver, spleen, stomach	bitter, natured
Nelumbinis Rhizomatis Nodus	Oujie	liver, lung, stomach	sweet, astringent, natured
Notoginseng Radix Et Rhizoma	Sanqi	liver, stomach	sweet, little bitter, temperature
Ophiopogonis Radix	Maidong	heart, lung, stomach	sweet, little bitter, little cold
Oroxyli Semen	Muhudie	lung, liver, stomach	bitter, sweet, cool
Oryzae Fructus Germinatus	Daoya	spleen, stomach	sweet, temperature
Panacis Majoris Rhizoma	Zhuzishen	liver, lung, stomach	bitter, sweet, little cold
Phragmitis Rhizoma	Lugen	lung, stomach	sweet, cold
Phyllanthifructus	Yuganzi	lung, stomach	sweet, sour, astringent, cool
Picrorhizae Rhizoma	Huhuanglian	liver, stomach, large intestine	bitter, cold
Pinelliae Rhizoma	Banxia	spleen, stomach, lung	pungent, temperature, toxic
Pinelliae Rhizoma Praeparatum	Fabanxia	spleen, stomach, lung	pungent, temperature

Piperis Fructus	Hujiao	stomach, large intestine	pungent, hot
Piperis Longi Fructus	Biba	stomach, large intestine	pungent, hot
Pogostemonis Herba	Guanghuoxiang	spleen, stomach, lung	pungent, little temperature
Polygonati Odorati Rhizoma	Yuzhu	lung, stomach	sweet, little cold
Polygoni Orientalis Fructus	Shuihonghuazi	liver, stomach	salty, little cold
Polygoni Tinctorii Folium	Liaodaqingye	heart, stomach	bitter, cold
Potentillae Discoloris Herba	Fanbaicao	liver, stomach, large intestine	sweet, little bitter, natured
Puerariae Lobatae Radix	Gegen	spleen, stomach, lung	sweet, pungent, cool
Puerariae Thomsonii Radix	Fenge	spleen, stomach	sweet, pungent, cool
Pulsatillae Radix	Baitouweng	stomach, large intestine	bitter, cold
Quisqualis Fructus	Shijunzi	spleen, stomach	sweet, temperature
Rabdosiae Rubescentis Herba	Donglingcao	lung, stomach, liver	bitter, sweet, little cold
Raphani Semen	Laifuzi	lung, spleen, stomach	pungent, sweet, natured
Rhapontici Radix	Loulu	stomach	bitter, cold
Rhei Radix Et Rhizoma	Dahuang	spleen, stomach, large intestine, liver, heart	bitter, cold
Santali Albi Lignum	Tanxiang	spleen, stomach, heart, lung	pungent, temperature
Scrophulariae Radix	Xuanshen	lung, stomach, kidney	sweet, bitter, salty, little cold
Semen Melo	Tianguazi	lung, stomach, large intestine	sweet, cold
Semiaquilegiae Radix	Tiankuizi	liver, stomach	sweet, bitter, cold
Setariae Fructus Germinatus	Guya	spleen, stomach	sweet, temperature
Siphonostegiae Herba	Beiliujinu	spleen, stomach, liver, gallbladder	bitter, cold
Smilacis Glabrae Rhizoma	Tufuling	liver, stomach	sweet, bland, natured
Sojae Semen Germinatum	Dadouhuangjuan	spleen, stomach, lung	sweet, natured
Sojae Semen Praeparatum	Dandouchi	lung, stomach	pungent, bitter, cool
Sophorae Tonkinensis Radix Et Rhizoma	Shandougen	lung, stomach	bitter, cold, toxic
Sophorae Flavescentis Radix	Kushen	heart, liver, stomach, large intestine, bladder	bitter, cold
Stachyuri Medulla Helwingiae Medulla	Xiaotongcao	lung, stomach	sweet, bland, cold
Stauntoniae Caulis Et Folium	Yemugua	liver, stomach	little bitter, natured
Stellariae Radix	Yinchaihu	liver, stomach	sweet, little cold
Swertiae Herba	Dangyao	liver, stomach, large intestine	bitter, cold
Tamaricis Cacumen	Xiheliu	heart, lung, stomach	sweet, pungent, natured
Taraxaci Herba	Pugongying	liver, stomach	bitter, sweet, cold
Tetrapanacis Medulla	Tongcao	lung, stomach	sweet, bland, little cold
Torreyae Semen	Feizi	lung, stomach, large intestine	sweet, natured
Trichosanthis Radix	Tianhuafen	lung, stomach	sweet, little bitter, little cold
Trichosanthis Fructus	Gualou	lung, stomach, large intestine	sweet, little bitter, cold
Trichosanthis Pericarpium	Gualoupi	lung, stomach	sweet, cold
Trichosanthis Semen	Gualouzi	lung, stomach, large intestine	sweet, cold
Trichosanthis Semen Tostum	Chaogualouzi	lung, stomach, large intestine	sweet, cold
Tsaoko Fructus	Caoguo	spleen, stomach	pungent, temperature

Typhonii Rhizoma	Baifuzi	stomach, liver	pungent, temperature, toxic
Vaccariae Semen	Wnagbuliuxing	liver, stomach	bitter, natured
Viticis Fructus	Manjingzi	bladder, liver, stomach	pungent, bitter, little cold
Vladimiriae Radix	Chuanmuxiang	spleen, stomach, large intestine, gallbladder	pungent, bitter, temperature
Zanthoxyli Pericarpium	Huajiao	spleen, stomach, kidney	pungent, temperature
Zanthoxyli Radix	Liangmianzhen	liver, stomach	bitter, pungent, natured, little toxic
Zingiberis Rhizoma	Ganjiang	spleen, stomach, kidney, heart, lung	pungent, hot
Zingiberis Rhizoma Praeparatum	Paojiang	spleen, stomach, kidney	pungent, hot
Zingiberis Rhizoma Recens	Shengjiang	lung, spleen, stomach	pungent, little temperature

Supplementary S5 The list of Chinese Patent Medicine of CVDs

Chinese patent medicine	Diseases of CVDs	Chinese patent medicine	Diseases of CVDs
Yangxinshu cappules	Angina	Fufang danshen pills	Angina pectoris
Yixintongmai particles	Angina	Maiguantong pills	Angina pectoris
Yixintong pills	Angina	Guanxindanshao pills	Angina pectoris
Yixin pellets	Angina	Guanxindanshen pills	Angina pectoris
Yinxingye pills	Angina	Guanxinjing capsules	Angina pectoris
Yufengningxin particles	Angina	Guanxinkang pills	Angina pectoris
Yuxintong capsules	Angina	Guanxinshutong capsules	Angina pectoris
Zhenxintong oral liquid	Angina	Guanxintai pellets	Angina pectoris
Zhengxintai capsules	Angina	Huangqishengmai particles	Angina pectoris
Zixinyin capsules	Angina	Huangyangning pills	Angina pectoris
Baoxinbao	Cardiodynia	Huoxin pellets	Angina pectoris
Buxinqi oral liquid	Cardiodynia	Jiruxin pills	Angina pectoris
Danling xinshu capsules	Cardiodynia	Jinyinsanqi capsules	Angina pectoris
Danlou pills	Cardiodynia	Jingzhiguanxin pills	Angina pectoris
Dengzhanhua particles	Cardiodynia	Jiuxinjin pellets	Angina pectoris
Dengzhanshengmai capsules	Cardiodynia	Jiuxin pellets	Angina pectoris
Dengzhanxixin injection	Cardiodynia	Kudiezi injection	Angina pectoris
Dunye guanxingning pills	Cardiodynia	Lingbaohuxin pills	Angina pectoris
Fuxin pills	Cardiodynia	Mailuotong particles	Angina pectoris
Guanxinkang pills	Cardiodynia	Maiping pills	Angina pectoris
Guanxintai pellets	Cardiodynia	Meiguo oral liquid	Angina pectoris
Huangqishengmai particles	Cardiodynia	Naoxinan capsules	Angina pectoris
Huangyangning pills	Cardiodynia	Naoxinqing pills	Angina pectoris
Huoxin pellets	Cardiodynia	Naoxintong capsules	Angina pectoris
Huoxuetongluo capsules	Cardiodynia	Nuodikang capsules	Angina pectoris
Jiuxinjin pellets	Cardiodynia	Qishenyisi drop pills	Angina pectoris
Jiuxin pellets	Cardiodynia	Sanqi guanxinning mixture	Angina pectoris
Kangfuchun oral liquid	Cardiodynia	Sanqi capsules	Angina pectoris
Kudiezi injection	Cardiodynia	Sanshenjiangzhi liquid	Angina pectoris
Maiping pills	Cardiodynia	Shanhaidan pills	Angina pectoris
Naoxinan capsules	Cardiodynia	Shexiangbaoxin pellets	Angina pectoris
Naoxinqing pills	Cardiodynia	Shexiang xintong ointment	Angina pectoris
Naoxintong capsules	Cardiodynia	Shenlongningxin capsules	Angina pectoris
Sanqi guangxinning mixture	Cardiodynia	Shenxiang suhe pellets	Angina pectoris
Sanshen jiangzhi fluid	Cardiodynia	Shuxin jiangzhi pills	Angina pectoris
Shexiang xintong ointment	Cardiodynia	Shuxin oral liquid	Angina pectoris
Shenxiang suhe pellets	Cardiodynia	Shuxiong pills	Angina pectoris
Shuxin jiangzhi pills	Cardiodynia	Shuangdan oral liquid	Angina pectoris
Shuangdan oral liquid	Cardiodynia	Shuangshenlong capsules	Angina pectoris
Suxiaoxintong aerosol	Cardiodynia	Tongmai ciwujia capsules	Angina pectoris
Tongqiaoyixin pills	Cardiodynia	Xinfukang capsules	Angina pectoris

Tongxinshu capsules	Cardiodynia	Xinli pellets	Angina pectoris
Xindakang pills	Cardiodynia	Xinling pellets	Angina pectoris
Xinfukang capsules	Cardiodynia	Xinnaokang capsules	Angina pectoris
Xinli pellets	Cardiodynia	Xinnaoning capsules	Angina pectoris
Xinling pellets	Cardiodynia	Xinnao qingruan capsules	Angina pectoris
Xinnaoqingruan capsules	Cardiodynia	Xinnao shutong capsules	Angina pectoris
Xinnaoshutong capsules	Cardiodynia	Xinning pills	Angina pectoris
Xintong oral liquid	Cardiodynia	Xinrong oral liquid	Angina pectoris
Xintongning drop pills	Cardiodynia	Xinshule pills	Angina pectoris
Xintongning aerosol	Cardiodynia	Xinshuning pills	Angina pectoris
Xiongdanjiuxin pellets	Cardiodynia	Xinshu oral liquid	Angina pectoris
Xuesaitong capsules	Cardiodynia	Xintongning drop pills	Angina pectoris
Xueshuangtong capsules	Cardiodynia	Xintongning spray	Angina pectoris
Yangxinshengmai particles	Cardiodynia	Xinwuyou pills	Angina pectoris
Yixinfumai particles	Cardiodynia	Xinxinshu capsules	Angina pectoris
Yixintongmai particles	Cardiodynia	Xinxuening pills	Angina pectoris
Yixin pellets	Cardiodynia	Xinyuan capsules	Angina pectoris
Yinxingye pills	Cardiodynia	Xingling particles	Angina pectoris
Yingxindan	Cardiodynia	Xiongdanjiuxin pellets	Angina pectoris
Zhengxinjiangzhi pills	Cardiodynia	Xuefuzhuyu capsules	Angina pectoris
Zhijiangning pills	Cardiodynia	Xuesaitong capsules	Angina pectoris
Ankangxinbao pellets	Angina pectoris	Xueshuangtong capsules	Angina pectoris
Baoxinbao	Angina pectoris	Yanghuosanqi pills	Angina pectoris
Bingqi pills	Angina pectoris	Yangxinshengmai particles	Angina pectoris
Buxinqi oral liquid	Angina pectoris	Yinaoning pills	Angina pectoris
Danhong solution	Angina pectoris	Yixinfumai particles	Angina pectoris
Danlou pills	Angina pectoris	Yixin capsules	Angina pectoris
Danqi pills	Angina pectoris	Yixinshu capsules	Angina pectoris
Danshen pills	Angina pectoris	Yixin tongmai particles	Angina pectoris
Danxiang glucose solution	Angina pectoris	Yixintong pills	Angina pectoris
Danxiongtongmai particles	Angina pectoris	Yixin pellets	Angina pectoris
Dengzhanhua particles	Angina pectoris	Yinxingye pills	Angina pectoris
Dengzhanshengmai capsules	Angina pectoris	Zhenxintong oral liquid	Angina pectoris
Dengzhanxixin capsules	Angina pectoris	Zhengxin jiangzhi pills	Angina pectoris
Dengzhanxixin injection	Angina pectoris	Zhengxintai capsules	Angina pectoris
Di'aoxinxuekang capsules	Angina pectoris	Zhijiangning pills	Angina pectoris
Dunye guanxinning pills	Angina pectoris	Zhusheyong danshen	Angina pectoris
Fufang danshen drop pills	Angina pectoris	Zixinyin capsules	Angina pectoris

Supplementary S6 The list of Chinese Patent Medicine of GIDs

Chinese patent medicine	Diseases of GIDs	Chinese patent medicine	Diseases of GIDs
Guhaisheng pills	Peptic ulcer	Fufang chenxiang wei pills	Chronic Gastritis
Haiganwei pills	Peptic ulcer	Fufang houtou particles	Chronic Gastritis
Hewei pills	Peptic ulcer	Fufang longmaluwei pills	Chronic Gastritis
Jianwei Yuyang pills	Peptic ulcer	Tianqi weitong particles	Chronic Gastritis
Jinlian Weishu pills	Peptic ulcer	Ganhaiweihai capsules	Chronic Gastritis
Kuidekang particles	Peptic ulcer	Hougu pills	Chronic Gastritis
Pingkui power	Peptic ulcer	Houtou jianweiling capsules	Chronic Gastritis
Qiangli Fengrujiangjiao pills	Peptic ulcer	Houtoujun pills	Chronic Gastritis
Shenjiao particles	Peptic ulcer	Jianwei xiaoyan particles	Chronic Gastritis
Weidekang particles	Peptic ulcer	Jinfo zhitong pills	Chronic Gastritis
Weisu particle	Peptic ulcer	Jinlian weishu pills	Chronic Gastritis
Weitengning pills	Peptic ulcer	Jiubiying weitong pills	Chronic Gastritis
Weitongshu pills	Peptic ulcer	Kuiyang pills	Chronic Gastritis
Weixiangning particles	Peptic ulcer	Kuiyangsan apsules	Chronic Gastritis
Weiyouxin pills	Peptic ulcer	Longqi weikang pills	Chronic Gastritis
Xiangyao Weian capsules	Peptic ulcer	Ludangshen ointment	Chronic Gastritis
Xueshan Weibao pills	Peptic ulcer	Nuanwei shule particle	Chronic Gastritis
Xinxu Weitong particle	Peptic ulcer	Piweishu pills	Chronic Gastritis
Yuanhe Zhengwei pills	Peptic ulcer	Quyuyiwei capsules	Chronic Gastritis
Danguixiang particles	Chronic atrophic gastritis	Saiweian capsules	Chronic Gastritis
Hewei Jiangni capsules	Chronic atrophic gastritis	Shenchai particle	Chronic Gastritis
Knagji Xiaoke pills	Chronic atrophic gastritis	Shenmeiyangwie particle	Chronic Gastritis
Moluo oral liquid	Chronic atrophic gastritis	Weidean pills	Chronic Gastritis
Quyu Yiwei capsules	Chronic atrophic gastritis	Weierning pills	Chronic Gastritis
Shenqi Jianwei particles	Chronic atrophic gastritis	Weian particles	Chronic Gastritis
Weierkang pills	Chronic atrophic gastritis	Weikang particles	Chronic Gastritis
Weilening pills	Chronic atrophic gastritis	Weikangling particles	Chronic Gastritis
Weileshu oral liquid	Chronic atrophic gastritis	Weile particles	Chronic Gastritis
Weilexin capsules	Chronic atrophic gastritis	Weinaian capsules	Chronic Gastritis
Weiwanshu particles	Chronic atrophic gastritis	Weining capsules	Chronic Gastritis
Wenweishu capsules	Chronic atrophic gastritis	Weisu particles	Chronic Gastritis
Xuhanweitong particles	Chronic atrophic gastritis	Weitongping particles	Chronic Gastritis
Yangwei paiticle	Chronic atrophic gastritis	Weitongshu pills	Chronic Gastritis
Yangweishu capsules	Chronic atrophic gastritis	Weixiangning particles	Chronic Gastritis
Yangyinqingwei particle	Chronic atrophic gastritis	Weixinshu capsules	Chronic Gastritis
Anwei capsules	Chronic Gastritis	Weiyankang capsules	Chronic Gastritis
Anzhong pills	Chronic Gastritis	Weiyouxin pills	Chronic Gastritis
Bilingweitong particles	Chronic Gastritis	Wenwei jiangni particles	Chronic Gastritis
Caoxiangweikang capsules	Chronic Gastritis	Xueshanweibao pellets	Chronic Gastritis
Chenxiang lubailu pills	Chronic Gastritis	Yangweishu capsules	Chronic Gastritis
Zhenhuang wei pills	Chronic Gastritis	Yiwei oral liquid	Chronic Gastritis

Zhengwie pills	Chronic Gastritis
